# Cellulase production, optimization and characterization from *Bacillus velezensis* 2H using carrot waste as a substrate; response surface methodology (RSM) and Artificial Neural Network (ANN) modeling

**DOI:** 10.3934/microbiol.2026032

**Published:** 2026-10-14

**Authors:** Iram Yasmeen, Muhammad Zubair Ali, Sri Rahayu, Hafiz Abdullah Shakir, Muhammad Khan, Yuliya Kulikova, Olga Babich, Muhammad Irfan, Trisanti Anindyawati

**Affiliations:** 1 Department of Biotechnology, University of Sargodha, Sargodha, Pakistan; 2 Biology Department, Faculty of Mathematics & Natural Sciences, Universitas Negeri Jakarta, Jakarta, Indonesia; 3 Institute of Zoology, University of the Punjab, New Campus, Lahore, Pakistan; 4 Research and Education Center of Industrial Biotechnologies, Immanuel Kant Baltic Federal University, Kaliningrad, Russia; 5 Research Center for Applied Microbiology, National Research and Innovation Agency, Jl. Raya Bogor KM 46, Cibinong, Bogor 16911, West Java, Indonesia

**Keywords:** cellulase, CMCase, carrot waste, FPase, RSM, ANN, bacterial strain, characterization, optimization

## Abstract

Lignocellulosic resources are naturally renewable, low-priced, and abundant. They comprise three kinds of polymers, cellulose, hemicelluloses, and lignin, bonded to each other with the help of covalent cross-linking and non-covalent forces. Cellulose is the world's most abundant organic material and the chief constituent of biomass energy. Moreover, production of calluses from *Bacillus valezensis* strain 2H was statistically optimized by substrate carrot in submerged fermentation using Response Surface Methodology (RSM) and Artificial Neural Network (ANN). The RSM and ANN were used to successfully optimize Carboxymethyl cellulase (CMCase) and Filter paper (FPase). Moreover, RSM was utilized for optimization of the target enzyme using a newly identified *Bacillus velezensis* strain. Plackett-Burman design (PBD) was adopted for screening of the most significant factors affecting cellulase production. Central composite design (CCD) was utilized to optimize enzyme production. Biochemical characterization revealed that both enzymes were optimally active under acidic conditions (pH 6.0), with the optimum temperature observed to be 60 °C, and stability was maintained up to 60 °C. The tested metal ions affected CMCase and FPase activities to varying degrees but did not enhance enzyme activity compared with the control. Similarly, the tested organic solvents generally decreased enzyme activities, although the extent of inhibition varied depending on the enzyme and solvent. R^2^ values for the CMCase were 95.43% and for FPase 99.58%, calculated by RSM analysis. R^2^ values for CMCase were 91.44% (Overall R = 0.95622), and for FPase were 53.70% (Overall R = 0.7328), calculated by ANN analysis. Our results showed that the RSM and ANN models provided different predictive performances for CMCase and FPase. This study elucidates the potential of *Bacillus velezensis* for efficient cellulase production under optimized submerged fermentation conditions.

## 1. Introduction

Lignocellulosic biomass is one of the significant and renewable sources of carbon that we can use to produce fuels, chemicals, and useful products in a sustainable way. Agro-industrial leftovers, such as fruit and vegetable scraps, crop stalks, straw, and other plant materials hold large amounts of cellulose and hemicellulose that can be turned into sugars that microbes can ferment into biofuels and biobased chemicals. The efficient use of lignocellulosic biomass is difficult because cellulose is inside a tough mix of hemicellulose and lignin. The hard structure of cellulose and the bonds between lignin and sugars makes it so that enzymes cannot easily reach the material, and the creation or release of compounds that block enzymes during pre-treatment can stop enzymes from breaking down the sugars [1]–[3].

The conversion of biomass into fermentable sugars usually begins with pretreatment and then goes to enzymatic hydrolysis. Pretreatment helps break down the structure of biomass. It does this by loosening the link between lignin and carbohydrates, making cellulose crystalline and moving lignin around, making more surface available for enzymes. Choosing the pretreatment is very important; if the pretreatment is too harsh, it can require a lot of energy and chemicals and can create substances that stop enzymes from working well later. Because of this research, biorefineries is moving toward ways that treat biomass, convert it, and process the products in a greener way instead of doing each step separately [2],[4]. One promising way is using eutectic solvents (DESs) and natural deep eutectic solvents (NaDESs). These liquids can be tuned to have properties and can make biomass easier to break down under gentler conditions [5],[6]. Studies by Cajnko and colleagues showed how NaDESs can help with extraction and biocatalysis. Bjelić and others reviewed how NaDESs can be used for parts of lignocellulose biorefineries, such as separating, catalyzing, and enzyme reactions [5],[6]. Teržan and team discussed catalytic and biocatalytic methods to turn biomass sugars into useful products, stressing the need for selective and sustainable steps after the main conversion [7],[8]. All these works showed that to get the value from biomass, we need good pretreatment and strong enzymes that work well under real process conditions.

Following pretreatment, enzymatic hydrolysis is a step in converting cellulose into soluble sugars. Cellulose is mostly made of D-glucose units that are arranged in crystalline and amorphous parts. Enzymatic hydrolysis of cellulose does not happen well with an enzyme. Instead, enzymatic hydrolysis needs the action of different glycoside hydrolases. Endoglucanases (EC 3.2.1.4) cut internal β-1,4-glycosidic bonds and create new chain ends. Cellobiohydrolases or exoglucanases then work on the ends of cellulose chains to release cellobiose and short cellodextrins. β-Glucosidases (EC 3.2.1.21) finally break down cellobiose and soluble cello‑oligosaccharides into glucose. The complementary actions of these enzymes build effects and are essential for efficient cellulose saccharification [3],[8]. The efficiency of hydrolysis is affected by enzyme properties such as activity, stability, adsorption, substrate specificity, and enzyme synergy and by substrate properties such as crystallinity, accessible surface area, lignin content, and soluble inhibitors [3].

The importance of enzyme synergy in turning lignocellulose into products has been known for many years. The enzymatic hydrolysis stage is one of the complicated parts of converting biomass into biological products. This is because the performance of the enzymes is greatly influenced by the chemical traits of the material they are working on as well as how the glycosyl hydrolases interact [8]. They also pointed out that making this process efficient means looking at the variety of enzymes, how stable they are, the activities of each individual enzyme, and how they work together; not just adding more enzymes. Thus, creating cellulase systems means finding enzymes that have different abilities to break down materials and that stay stable under the conditions seen during biomass processing.

Studies continue to show how important it is to find and improve microbial cellulase enzymes. A genome-wide analysis of *Bacillus velezensis* SSF6 revealed genes related to carbohydrate-active enzymes. This strain showed the ability to break down cellulose with activities, from endoglucanase, exoglucanase, and β-glucosidase. These results suggest that *Bacillus velezensis* could be a source of enzymes that degrade lignocellulose [9]. Another strain, *Bacillus velezensis* Z2.6, was discovered as a cellulolytic bacterium. Its genome revealed genes involved in cellulose breakdown, including those coding for GH5 endo-1,4-β-D-glucanase and GH1 β-glucosidase. When researchers used experimental designs to optimize fermentation conditions, they found that culture settings greatly affect how much cellulase is produced [10]. These results are important because they show that finding only a strain is not enough. To obtain enzyme preparations that work well in real-world biomass conversion, we must carefully optimize how the enzymes are produced.

The qualities of the enzymes that are produced are equally important. Processing lignocellulose in the industry can reveal that enzymes can keep their catalytic power at higher temperatures because a higher temperature can make the substrate dissolve better, lower the thickness of the liquid, help material move faster, and lower the chance of microbes taking over. However, the power of enzymes and enzyme structure can fall quickly when the temperature or acidity is not right. For this reason, enzymes that stay stable at temperatures and that can handle different acidity levels are very interesting for industrial use. Research has revealed traits in cellulases that come from *Bacillus* bacteria. For instance, a cellulase made by *Bacillus velezensis* MRC 5958 works best at about 55 °C and pH 7.0, and enzymes from that strain stay stable over a temperature range from 30 to 70 °C [11]. That strain can also make cellulase from lignocellulosic materials, such as alkali‑treated sugarcane bagasse and rice waste. In a way, an endo-1,4-β-glucanase from *B. Velezensis* H1 works at 60 to 65 °C and retains much activity at high temperatures while resisting high salt and releasing cellulose pieces from farm waste [12]. A thermostable cellulase-producing *Bacillus licheniformis* strain taken from soil shows that heat-loving bacteria can be good sources of cellulases for the industry [13]. These studies show that bacterial cellulases can give mixes of power, heat tolerance, and ability to work with different materials.

Optimizing microbial enzyme production is another part of making cellulase processes affordable. The mix of the culture and the conditions that the culture grows in, such as the carbon and nitrogen sources, the pH, the temperature, the length of incubation, the amount of inoculum, and any added minerals, can have an effect on cellulase synthesis. Scientists have been using optimization methods instead of testing one factor at a time. For example, cellulase production by *Bacillus cereus* A49 was optimized with response surface methodology (RSM), showing that statistical methods can identify how different fermentation variables interact [14]. In this way, work with *Bacillus tequilensis* revealed that response-surface methodology (RSM) could greatly raise cellulase production and that the enzyme produced works on many different substrates [15]. These discoveries support the idea that systematic experiments and computer calculations can improve enzyme production for cellulase.

Despite these advances, a number of challenges exist. Many cellulases that have been reported show activity only within a narrow temperature and pH range. The link between how enzymes are produced, their biochemical traits, their stability, and how well they work on real lignocellulosic materials is not always fully understood. Moreover, laboratory tests often use soluble model substrates like carboxymethyl cellulose. In contrast, real biomass conversion needs enzymes that can work well with partly stubborn lignocellulosic materials. Therefore, finding microorganisms in less studied environments, improving how we produce and test enzymes, and studying their biochemistry in detail are all needed to broaden the set of sturdy cellulases. *Bacillus* species in particular are attractive because they grow fast, produce enzymes outside the cell, are easy to manipulate, and can make enzymes that work over a wide range of conditions [16].

In this study, we focused on finding and recognizing a type of cellulolytic *Bacillus* strain, making sure that its endoglucanase production is as good as possible. We looked at samples to identify bacteria that can break down cellulose. These bacteria were then checked for their shape and chemical properties. They were also identified using a method that looks at the 16S gene. The bacteria that were found were named *Bacillus velezensis*. Moreover, to make endoglucanase, we used a method called Plackett-Burman design (PBD) to determine which factors matter the most. We then used a RSM and artificial neural networks (ANNs) to enhance the process. We also looked at the properties of the enzyme to see how stable it is and how well it can work in breaking down plant material. By using all these steps, from finding the bacteria to making the enzyme better, we aim to learn more about how bacteria make endoglucanase and to help create tools for turning plants into useful products in a green way.

## 2. Materials and methods

### 2.1. Microbial strain

In this study, *Bacillus velezensis* strain 2H was isolated from a soil sample collected from the local region of Shahpur district, Sargodha, Punjab, Pakistan. To differentiate the cellulase producing bacterial strain, the sample dilution was cultured on Carboxymethyl cellulose (CMC) agar plates (nutrient agar containing 1% CMC), incubated at 37 °C for 24 hours. Only well-formed bacterial colonies were taken for the next step. The target strain was purified using the repeated plate streaking method. Genomic DNA was extracted following international standard protocols, and the 16S ribosomal RNA sequencing technique was used for molecular identification of the selected strain. In this research, the molecular identification results identified it as *Bacillus velezensis* strain 2H, which was further explored for the production of cellulase enzymes.

### 2.2. Substrate collection and media preparation

Carrots were collected from local fruit shops, household waste, fruit processing industries, and juice point waste in the Shahpur district, Sargodha, Punjab, Pakistan. Polythene bags were used for carrying the collected carrots to the laboratory. Carrots were washed with distilled water and dried in the oven overnight at 60 °C and sterilized using an autoclave (121 °C, 15 min, and 15 psi). After that, the sterilized carrots were dried to obtain fine powder and preserved in sterile, airtight jars. Media were prepared using carrot substrate, yeast extracts, and peptone as a nitrogen source, CaCl_2_ as a mineral, NaCl as salt, and other additional chemicals were added to obtain maximum production of the target enzyme [17].

### 2.3. Inoculum preparation

*Bacillus velezensis* strain 2H was added to sterile nutrient broth using a sterile inoculum loop from nutrient agar plates, and the sterile nutrient agar vials were incubated at 37 °C for 24 hours. The next day, we obtained highly turbid nutrient broth, which showed maximum microbial growth inside the vial. This turbid suspension, having OD_600_ = 0.04, was used as a source of inoculum for further experiments. In the next step, inoculation was performed under highly sterile conditions under laminar airflow to avoid any kind of contamination problems. An inoculum volume of 200 µL, which is roughly 1% of the experimental culture volume, was carefully moved into each experimental flask. The same inoculum volume and the same incubation conditions were kept for all runs so that the differences that could arise from inoculation procedures were kept as small as possible. The inoculated flasks were placed in the incubation shaker at 37 °C for 24 hours. Highly controlled conditions were provided under the incubation shaker, and the results showed maximum bacterial growth for the carrots [18].

### 2.4. Fermentation technique

In cellulase enzyme production, the submerged fermentation strategy was used with *Bacillus velezensis* strain 2H. For cellulase enzyme production, the basic fermentation medium containing 1% substrate was added to the 250 mL flask. Under international standards, it was sterilized through autoclave conditions (121 °C, 15 min, and 15 psi). After sterilization, the flasks were placed at room temperature to cool, and 1% fresh inoculum was added to the sterilized medium. The inoculated flasks were placed on an incubation shaker at 35 °C with a shaking speed of 120 rpm for 24 hours. After incubation, the produced enzymes were collected via centrifugation (10,000 rpm, 20 min) to obtain the supernatant for Carboxymethyl cellulase (CMCase) and Filter paper (FPase) enzyme assays [19].

#### 2.4.1. CMCase and FPase assays

CMCase and FPase were performed using the DNS method. Carboxymethyl cellulase activity was obtained by incubating 0.5 mL crude enzyme with 0.5 mL CMC (1%) for the time period of 30 minutes in a water bath (50 °C). In the same way, filter paper activity was obtained by incubating filter paper strips 1 × 6 cm dipped in 0.5 mL sodium citrate buffer (0.05 M, pH 5.0) with 500 µL crude enzyme for 30 minutes at 50 °C. After incubation, the cellulolytic activity was stopped by adding 1.5 mL DNS, which was then incubated in boiling water for 10 minutes. After that, the optical density was measured at 540 nm using a spectrophotometer. Each sample was run in duplicate and compared with a standard containing 1% glucose. A glucose calibration curve was used to measure the reducing sugars.

### 2.5. Process parameter optimization

To optimize the culture condition, one factor at a time was used, and the PBD was used to screen significant nutritional parameters. After screening of the most remarkable factors, these were further optimized using a CCD of the RSM. All the above statistical optimizations of these factors were performed to maximize cellulase activity.

#### 2.5.1. Nutritional factors screening via PBD design

PBD is considered the most important tool for screening and identifying significant factors affecting enzyme production. A total of 8 variables were studied, including X1 = yeast extract, X2 = KH_2_PO_4_, X3 = NaCl, X4 = MgSO_4_, X5 = K_2_HPO_4_, X6 = FeSO_4_, X7 = Peptone, and X8 = CaCl_2_. These variables were studied over 12 runs with low and high ranges, as shown in the Table 1 [20].

**Table 1.** Nutritional parameters used to formulate the Plackett-Burman Design.

Symbol	Parameter	High value (+1)	Low value (−1)
X1	Yeast extract	0.2	0.002
X2	KH_2_PO_4_	0.02	0.004
X3	NaCl	0.1	0.002
X4	MgSO_4_	0.04	0.002
X5	K_2_HPO_4_	0.02	0.002
X6	FeSO_4_	0.02	0.002
X7	Peptone	0.1	0.002
X8	CaCl_2_	0.01	0.002

#### 2.5.2. Optimization by RSM

The optimization technique of RSM was used to maximize target enzyme production. The screened variables identified through PBD was further optimized by CCD, and the response (enzyme production) was obtained through second order polynomial regression [21].

#### 2.5.3. Artificial Neural Network

Development of an ANN model using the Neural Network Toolbox of MATLAB R2015a (The Math Works Inc., Natick, MA, USA) was carried out to optimize the production of CMCase and FPase. This ANN model had three input parameters (Yeast extract, FeSO_4_, Peptone), hidden layers, and output (enzyme activity) layers with a (3-n-1) topological arrangement. The optimal number of neurons in the hidden layers was obtained by training different networks based on minimum root mean square error (RMSE). The backpropagation learning rule known as the Levenberg-Marquardt backpropagation (trainlm) algorithm was used in this study for training purposes. The training set was divided in such a way that 70% of the data belonged to the training subset, 15% to the validation subset, and another 15% to the test subset. Performance criteria included the determination coefficient (R^2^) and RMSE. The ANN architecture is depicted in Figure 1.

**Figure 1.**
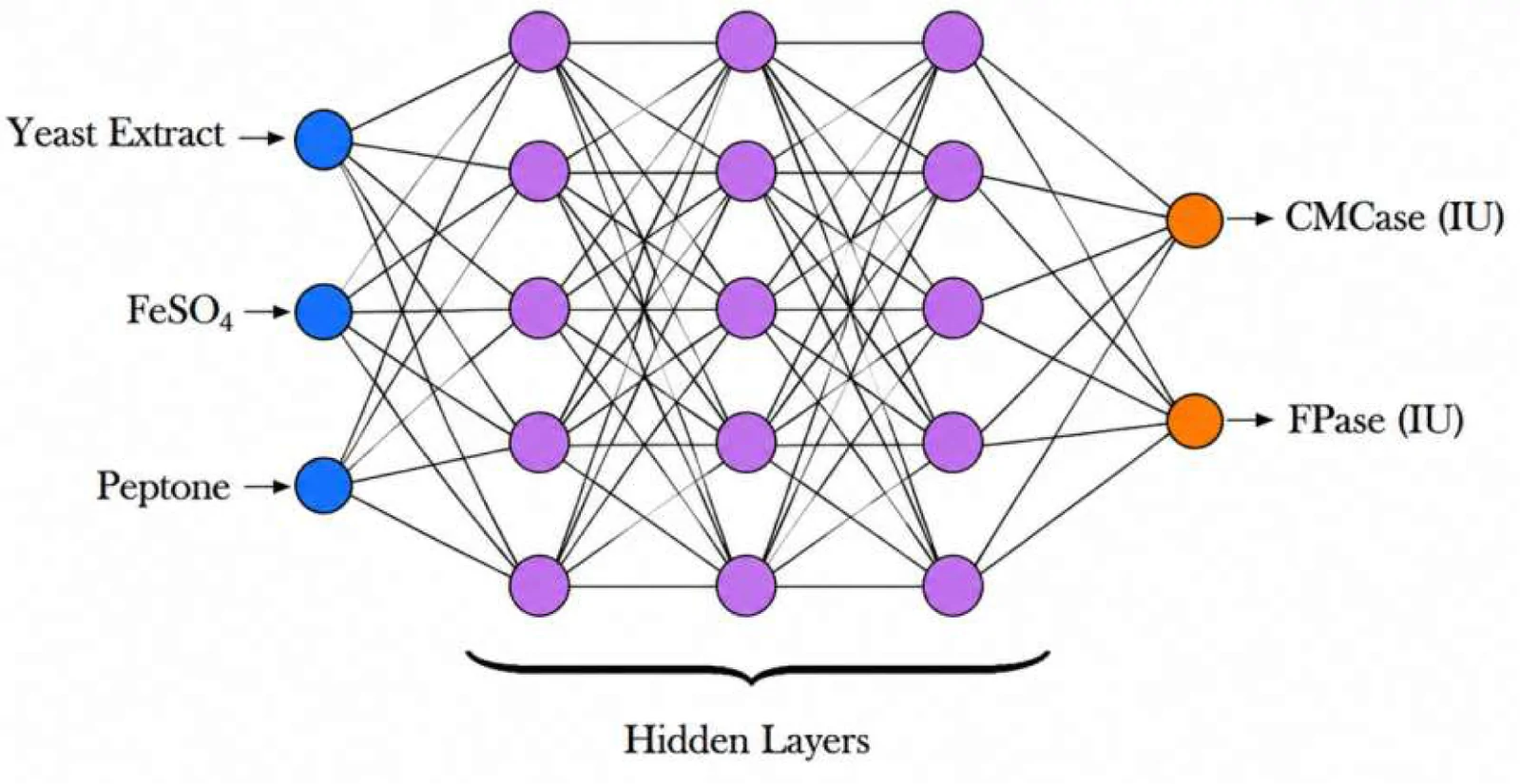
ANN architecture followed during CMCase and FPase optimization.

## 3. Characterization of the cellulolytic enzyme

### 3.1. Temperature

The activity and stability of the crude cellulolytic enzyme were examined by incubating the crude enzyme at varying temperatures. Its cellulose-degrading stability was examined by incubating crude cellulolytic enzyme + substrate (FPase and CMCase) separately at 20 °C, 30 °C, 40 °C, 50 °C, 60 °C, 70 °C, 80 °C, and 90 °C for a period of 30 minutes using a water bath at 2 h. Similarly, its activity was studied by keeping crude cellulase separate at the above-mentioned temperature range for a period of 30 minutes. Moreover, pre-incubated enzyme activity was examined using CMCase and FPase assays. In the case of enzyme stability, its activity was expressed as residual or relative enzyme activity.

### 3.2. pH

Cellulolytic enzyme stability and activity were analyzed by incubating the crude enzyme in different pH buffers such as 4.0, 5.0, 6.0, 7.0, 8.0 and 9.0 during the enzyme assay. The mentioned pH buffers were used to prepare CMC and filter paper substrates. To check the cellulolytic activity, the crude cellulase enzyme was added to each buffer separately, and CMCase and FPase assays were performed as per standard assay procedures. In the case of crude enzyme stability, the enzyme was first pre-incubated in buffers of the above-mentioned pH values for a period of 2 hours, and later, a normal enzyme assay was performed for CMCase and FPase assays. These effects on enzyme stability were indicated in residual or relative enzyme activities, while the effect on enzyme activity was measured in unit enzyme activity (Umol/minutes).

### 3.3. Metal ions

To check the effect of metal ions on crude cellulolytic enzyme activity, we used different metal ion solutions, including EDTA, KCl, ZnSO_4_, MnSO_4_, CuSO_4_, NaCl, MgSO_4,_ and FeSO_4_, at 5 mM concentrations. Subsequently, the residual and relative enzyme activities were checked by incubating the crude enzyme separately in each metal ion solution for 2 hours. Thereafter, normal enzyme assays for CMCase and FPase were performed and expressed in residual enzyme activity [22].

### 3.4. Solvents

The effect of different solvents on enzyme activity was analyzed by pre-incubating the crude cellulase enzyme separately in each solvent. Different solvents used for this purpose included Methanol, Butanol, Ethanol, Isopropanol, Acetone, and Chloroform. In this experiment, a standardized screening concentration of 5 mM was used to check their effect on enzyme stability and activity. After pre-incubation, a standard enzyme assay was performed for the CMCase and FPase assays. The results for enzyme activity were expressed as residual or relative enzyme activity.

### 3.5. Effect of surfactants and detergents

The effects of selected surfactants on CMCase and FPase activities were evaluated using Tween-80, Tween-20, Triton X-100, and SDS. Each surfactant was tested under the same experimental conditions used for the enzyme stability studies. The crude enzyme was incubated separately with the tested surfactants for the defined treatment period, after which residual CMCase and FPase activities were determined using the standard enzyme assay. The activity measured in the absence of the surfactant was considered the control, and the residual enzyme activity was expressed as a percentage of the control activity. The effects of commercially available detergents were also evaluated using Brite, Surf Excel, Express, and Tap under the experimental conditions described for the detergent assay. The residual CMCase and FPase activities were subsequently determined using the standard assay procedure.

### 3.6. Thermodynamics

Thermodynamic parameters such as enthalpy and entropy were analyzed based on crude cellulolytic enzyme activity at varying temperatures (20–70 °C). The activation energy (Ea) for FPase and CMCase was estimated using the Arrhenius plot method and was used further to calculate other thermodynamic parameters like enthalpy change (∆H) and entropy (∆S) [23]. The mathematical expressions used for evaluation of all these parameters are described below:

ΔG=−RT×LN (activity)

Ea=−Slope×R

ΔH‡=Ea−RT

ΔS=Intercept×R

### 3.7. Statistical analysis

In this research, all experiments were run in duplicate, and the mean values were used for further analysis. Graphical data were represented using Excel 2019, and further statistical analysis was performed using Minitab and MATLAB software.

## 4. Results and discussions

### 4.1. Molecular identification and PBD analysis

The bacterial strain was identified using the 16S ribosomal RNA sequencing technique, and genomic DNA was extracted in accordance with international standard methods. The forward primer 5′-AGAGTTTGATCMTGGCTCAG-3′ and the reverse primer 5′-RGYTACCTTGTTACGACTT-3′ were the sequencing primers employed in this investigation. The *Bacillus velezensis* strain was identified through molecular identification, and its enzyme production was further studied. The sequence having accession number of PX736032 was submitted and is available in the National Center for Biotechnology Information (NCBI).

In addition to strain identification analysis, enzyme production optimization evaluation was performed using the RSM technique [24]. The PBD was used to screen the most prominent parameters that affected cellulolytic enzyme production in our target strain [25]. The PBD is a category of statistical two-level factorial designs that is considered a standard approach to screen significant factors. There were eight parameters studied, including X1 = yeast extract, X2 = KH_2_PO_4_, X3 = NaCl, X4 = MgSO_4_, X5 = K_2_HPO_4_, X6 = FeSO_4_, X7 = Peptone, and X8 = CaCl_2_. These factors were studied with varying degrees of their concentrations, as demonstrated in code values such as maximum (+1) and minimum (−1), as shown in Table 1. These parameters were investigated using 12 experimental runs for the CMCase and FPase assays. Moreover, these experiments were run in duplicate to avoid mishandling. The results of the cellulolytic enzymes were demonstrated in unit enzyme activity (IU/mL/min) for the CMCase and FPase assays, as shown in Table 2.

**Table 2.** Plackett-Burman design was used for the variables to screen prominent nutritional factors for the cellulolytic enzyme production using the *Bacillus velezensis* 2H strain in submerged fermentation.

N	X1	X2	X3	X4	X5	X6	X7	X8	CMCase (IU/mL/min)	FPase (IU/mL/min)
1	+1	+1	+1	+1	+1	+1	+1	+1	167.3	123.33
2	−1	+1	−1	+1	+1	−1	−1	−1	168.37	129.5
3	−1	−1	+1	−1	+1	+1	+1	−1	169.13	57.96
4	+1	−1	−1	+1	−1	+1	+1	+1	168.37	123.33
5	−1	+1	−1	−1	+1	−1	+1	+1	153.83	97.98
6	−1	−1	+1	−1	−1	+1	−1	+1	150.31	113.83
7	−1	−1	−1	+1	−1	−1	+1	−1	192.4	106.1
8	+1	−1	−1	−1	+1	−1	−1	+1	101.97	113.82
9	+1	+1	−1	−1	−1	+1	−1	−1	121.53	78.07
10	+1	+1	+1	−1	−1	−1	+1	−1	101.2	84.65
11	−1	+1	+1	+1	−1	−1	−1	+1	106.01	113.54
12	+1	−1	+1	+1	+1	−1	−1	−1	116.33	118.97

For CMCase, the maximum enzyme production was achieved in experiment number 7 (192.4 IU/mL/min), while for FPase, the maximum enzyme production was achieved in experiment number 2 (129.5 IU/mL/min). In another study, researchers found the highest results led to screening the most significant variables using CCD. Moreover, Pareto chart analysis, performed as shown in Figure 2, indicated that Yeast extract, FeSO_4_, and peptone were significant variables for cellulase production, and these findings were in line with published studies [27]. The screened variables were further optimized through the CCD and ANN.

**Figure 2.**
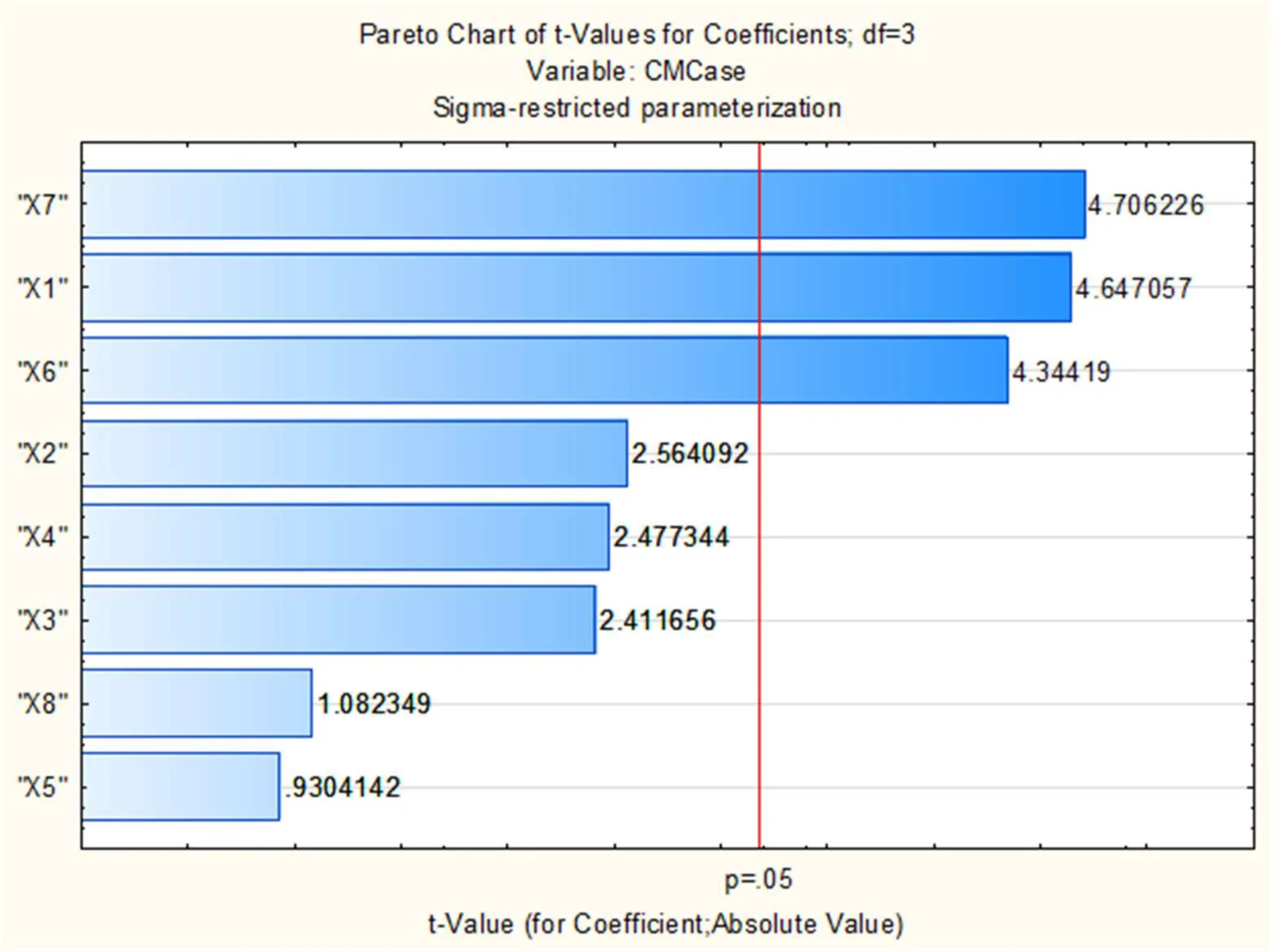
Pareto chart demonstrating the most significant variables for FPase and CMCase production.

### 4.2. CCD and ANN optimization evaluation

Three variables, including X1 = Yeast extract, X6 = FeSO_4_, and X7 = Peptone, were screened via PBD. The large impact of yeast extract and peptone came from their role as nitrogen sources that the cell uses to secrete cellulase [28],[29]. The effect of FeSO_4_ showed that small amounts of metal can change how much cellulase is produced, but the best yield differs for each *Bacillus* strain [30],[31]. Moreover, to optimize the concentrations of these prominent factors, the CCD was used, as shown in Table 3.

The results showed that maximum enzyme activity for CMCase (147.6 IU/mL/min) was recorded at 1.25% yeast extract, 1% FeSO_4_, 0.5% peptone, pH of 6.0, and temperature at 50 °C. Likewise, maximum enzyme activity for FPase (132.4 IU/mL/min) was recorded at 0.09% yeast extract, 0.65% FeSO_4_, and 0.3% Peptone. Contour plots were created to show the interaction effects of these variables on enzyme production (Figure 3).

**Table 3.** CCD design for the optimization of cellulolytic enzyme production.

No.	Yeast extract	FeSO_4_	Peptone	CMCase (IU/mL/min)			FPase (IU/mL/min)		
				Observed	Predicted	Residual	Observed	Predicted	Residual
1	0.01	0.1	0.5	141.6000	142.6374	−1.03736	82.7000	81.1243	1.57568
2	0.505	0.055	0.255	139.4000	139.9842	−0.58417	100.1000	100.2876	−0.18668
3	0.505	0.055	0.255	139.3000	139.9842	−0.68417	100.1000	100.2876	−0.18668
4	0.01	0.01	0.01	139.4000	138.7737	0.62633	92.5000	91.6733	0.82674
5	0.01	0.01	0.5	147.6000	148.9140	−1.31403	70.2000	71.9340	−1.73397
6	1	0.01	0.01	140.8000	140.6077	0.19230	93.9000	95.5585	−1.65853
7	1	0.1	0.01	139.5000	139.0310	0.46896	106.1000	104.4489	1.65112
8	−0.32749	0.055	0.255	145.8000	145.2727	0.52734	88.3000	88.3131	−0.01309
9	0.505	−0.02	0.255	139.1000	138.6194	0.48061	93.8000	92.6894	1.11061
10	0.505	0.055	0.255	139.4000	139.9842	−0.58417	100.7000	100.2876	0.41332
11	0.505	0.055	0.667	141.6000	139.1409	2.45910	98.3000	98.0800	0.21997
12	0.505	0.055	0.255	141.6000	139.9842	1.61583	100.1000	100.2867	−0.18668
13	0.505	0.055	0.255	139.9000	139.9842	−0.08417	100.3000	100.2867	0.01332
14	1	0.01	0.5	131.1000	132.0981	−0.99807	132.4000	131.7692	0.63077
15	0.01	0.1	0.01	128.6000	128.4470	0.15300	126.4000	127.1136	−0.71361
16	0.505	0.055	−0.157	133.1000	134.3640	−1.26399	106.2000	106.3028	−0.10281
17	0.505	0.055	0.255	140.1000	139.9842	0.11583	100.4000	100.2867	0.11332
18	1	0.1	0.5	133.1000	134.5714	−1.47140	113.5000	114.4096	−0.90958
19	1.33749	0.055	0.255	140.7000	140.0322	0.6676	119.7000	119.5698	0.13025
20	0.505	0.130	0.255	132.7300	132.0155	0.71448	106.9000	107.8934	−0.99345

**Figure 3.**
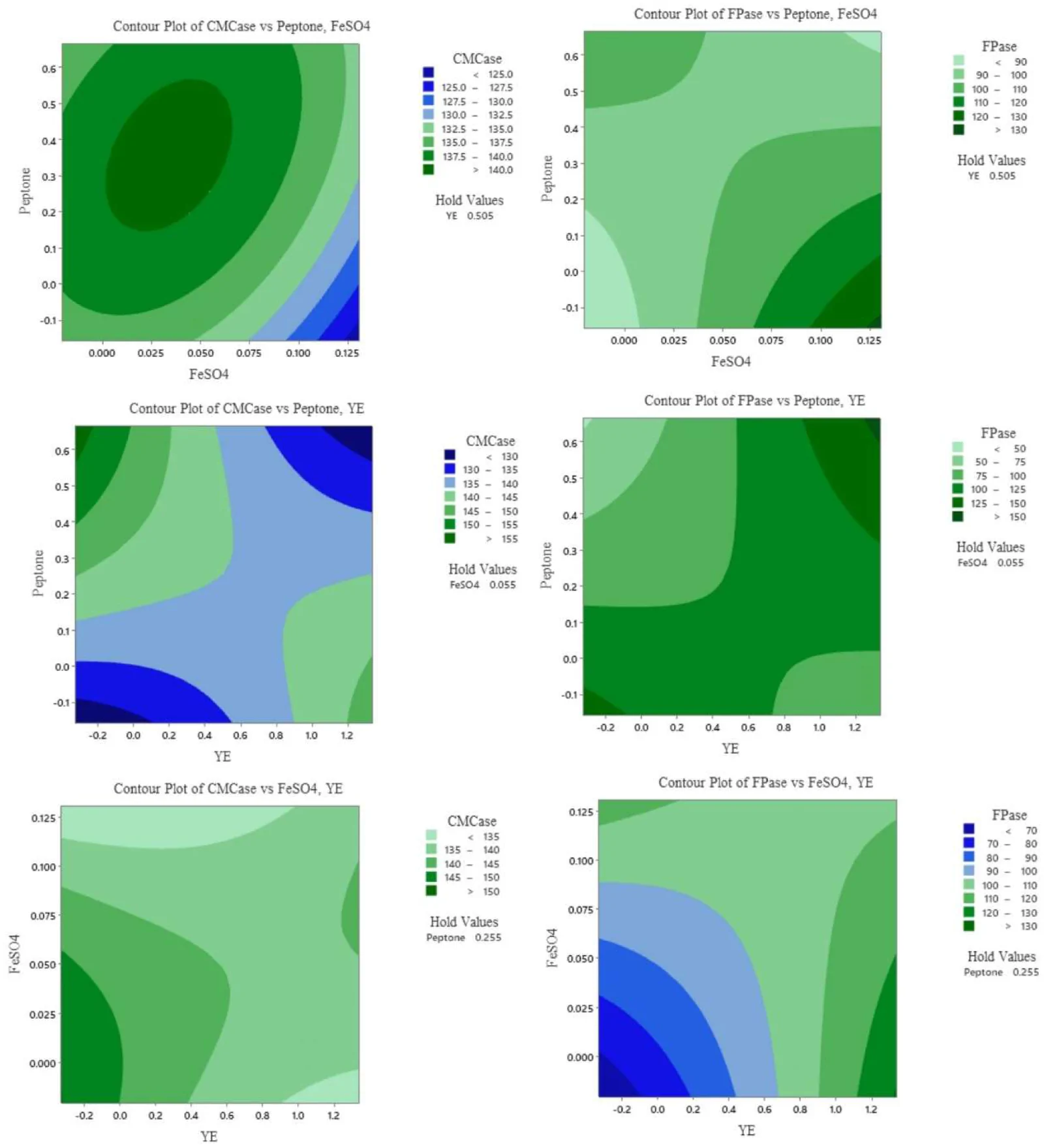
Contour plots indicating the interaction effects of different parameters on CMCase and FPase activities.

Moreover, these parameters were statistically analyzed via ANOVA, as shown in Tables 4 and 5. The P and F values showed the proposed model's significance. Moreover, multiple regression values (R^2^) for CMCase and FPase demonstrated great variability in CMCase and FPase production. Likewise, the correctness of the obtained results was shown by the proximity of values between the adjusted R^2^ and predicted multiple regression R^2^ for CMCase and FPase; if the regression R^2^ score was near 100%, it indicated a high correlation between experimental and predicted values. In this study, the R^2^ value for CMCase was 95.43%, which demonstrated the fitness of the selected model. Similarly, the R^2^ value for FPase was 99.58%, which was also a significant number but comparatively higher than that for CMCase. The high R^2^ values therefore confirmed the suitability of CCD for predicting cellulase production, consistent with other *Bacillus* optimization studies using RSM [32].

The CCD model examined the quadratic and interaction effects of these three variables within the defined experimental region. As a result, the CCD response showed how the fitted response surface behaved in the chosen factor space rather than simply optimizing the highest individual PBD run. The difference between the PBD response and the CCD response should not be seen as a failure of the optimization procedure.

**Table 4.** Evaluation of variance (ANOVA) for the most significant factors affecting CMCase activity.

Source	Df	Adj SS	Adj MS	F-value	P-value
Model	9	407.883	45.320	23.21	0.000
Linear	3	113.337	37.779	19.35	0.000
Yeast extract	1	33.149	33.149	16.97	0.002
FeSO_4_	1	52.643	52.643	26.96	0.000
Peptone	1	27.545	27.545	14.10	0.004
Square	3	74.152	24.717	12.66	0.001
Yeast exteact × yeast extract	1	12.826	12.826	6.57	0.028
FeSO_4_ × FeSO_4_	1	39.232	39.232	20.09	0.001
Peptone × Peptone	1	18.814	18.814	9.63	0.011
2-Way interaction	3	220.394	73.465	37.62	0.000
Yeast extract × FeSO_4_	1	38.281	38.281	19.60	0.001
Yeast extract × Peptone	1	173.911	173.911	89.05	0.000
FeSO_4_ × Peptone	1	8.201	8.201	4.20	0.068
Error	10	19.529	1.953		
Lack-of-fit	5	15.754	3.151	4.17	0.071
Pure error	5	3.775	0.755		
Total	19	427.412			

**Table 5.** Evaluation of variance (ANOVA) for the most significant factors affecting FPase activity.

Source	Df	Adj SS	Adj MS	F-value	P-value
Model	9	3830.99	425.67	266.56	0.000
Linear	3	1539.97	513.32	321.45	0.000
YE	1	1179.31	1179.31	738.50	0.000
FeSO_4_	1	279.04	279.04	174.74	0.000
Peptone	1	81.62	81.62	51.11	0.000
Square	3	28.84	9.61	6.02	0.013
YE × YE	1	24.06	24.06	15.07	0.003
FeSO_4_ × FeSO_4_	1	0.00	0.00	0.00	0.996
Peptone × Peptone	1	6.54	6.54	4.09	0.071
2-Way Interaction	3	2262.18	754.06	472.20	0.000
YE × FeSO_4_	1	352.45	352.45	220.71	0.000
YE × Peptone	1	1565.20	1565.20	980.15	0.000
FeSO_4_ × Peptone	1	344.53	344.53	215.75	0.000
Error	10	15.97	1.60		
Lack-of-Fit	5	15.68	3.14	54.38	0.000
Pure Error	5	0.29	0.06		
Total	19	3846.96			

An ANN is an advanced AI-based method primarily used for application-specific purposes [33]. The network processing unit, which is the input layer, receives the input vector. This is the initial step of feed forward network training [34]. Repeatedly, this method teaches the network to yield a different output during training. These networks produce outputs that are then compared with the desired outputs. Moreover, a network is changed after a comparison of output and target if its output is not equal to the target. Ultimately, the objective of training patterns is to minimize the global error. Furthermore, changing the weights and biases is how each method tries to reduce the global error.

The idea of using backpropagation to train a neural network with the Levenberg-Marquardt algorithm is being discussed [35]. The ANN model was created, and production conditions were optimized using MATLAB's neural network toolbox. Moreover, the 3-5-1 topology with the trainlm algorithm was employed [36]. Additionally, ANN includes an input layer with a fixed number of neurons, one output neuron, and one hidden layer [37].

The data was divided into three parts: 70% for training, 15% for validation, and 15% for testing [38]. Fitness of training (a), validation, test, and all data sets in the ANN model for CMCase optimization, (b) Error histogram, and (c) Training stat plot are shown in Figure 4. Fitness of training, validation, test, and all data sets in the ANN model for FPase optimization, (b) Error histogram, and (c) Training stat plot are shown in Figure 5. The error histogram indicates that most of the errors were near zero, showing a high level of prediction accuracy and low bias in the model output in the graph. A high level of close similarity between experimental and predicted values of the model is shown through close parallelism of lines with maxima on the lines, indicating the efficiency of the model in capturing the relationship between input and output values and therefore model accuracy and learning ability [32]. Similarly, coefficient of determination (R^2^) was equal to 91.44% and for 53.50% for CMCase and FPase, respectively, which were equal to Overall R values of 0.95622 for CMCase and 0.7328 for FPase, given in the regression plots. Moreover, the R values for training, validation, and testing of CMCase were 0.96892, 0.98883, and 0.98332, respectively, and for FPase were 0.80983, 0.11155, and 0.12403, respectively. Lower RMSE values also indicated good predictive performance; this meant that the 70% CCD data, which were used for training the ANN model, trained the model significantly. On the other hand, the 15% CCD data, which were used for testing purposes, also provided optimized results; on the basis of the trained data, the model tested the 15% values effectively, as shown by the testing R value.

This was especially the case for CMCase. For FPase, the results were worse when predicted by ANN due to a noisy or small dataset.

**Figure 4.**
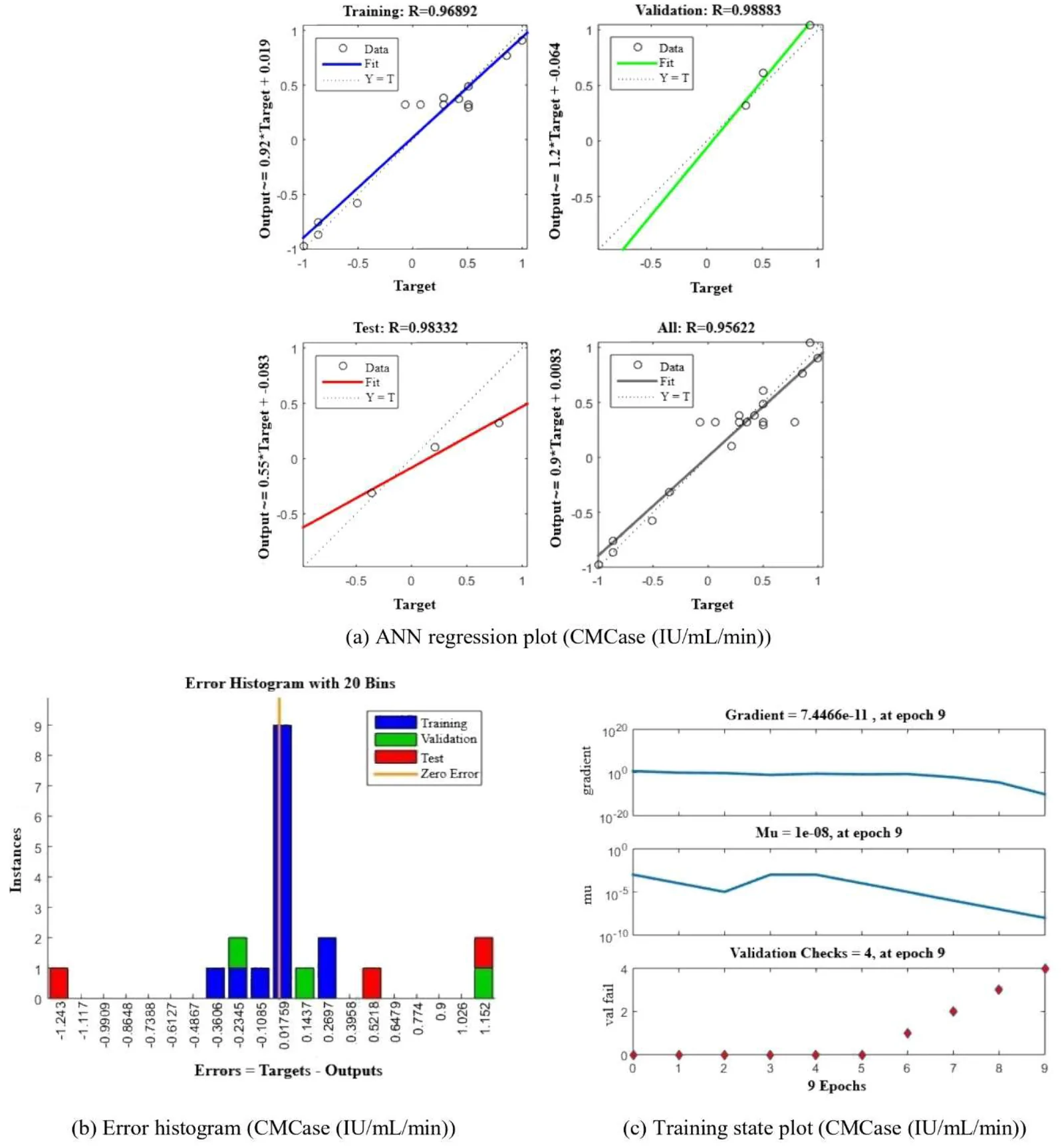
(a) Fitness of training, validation, test, and all data sets in the ANN model for CMCase optimization, (b) Error histogram, and (c) Training state plot (CMCase (IU/mL/min)).

**Figure 5.**
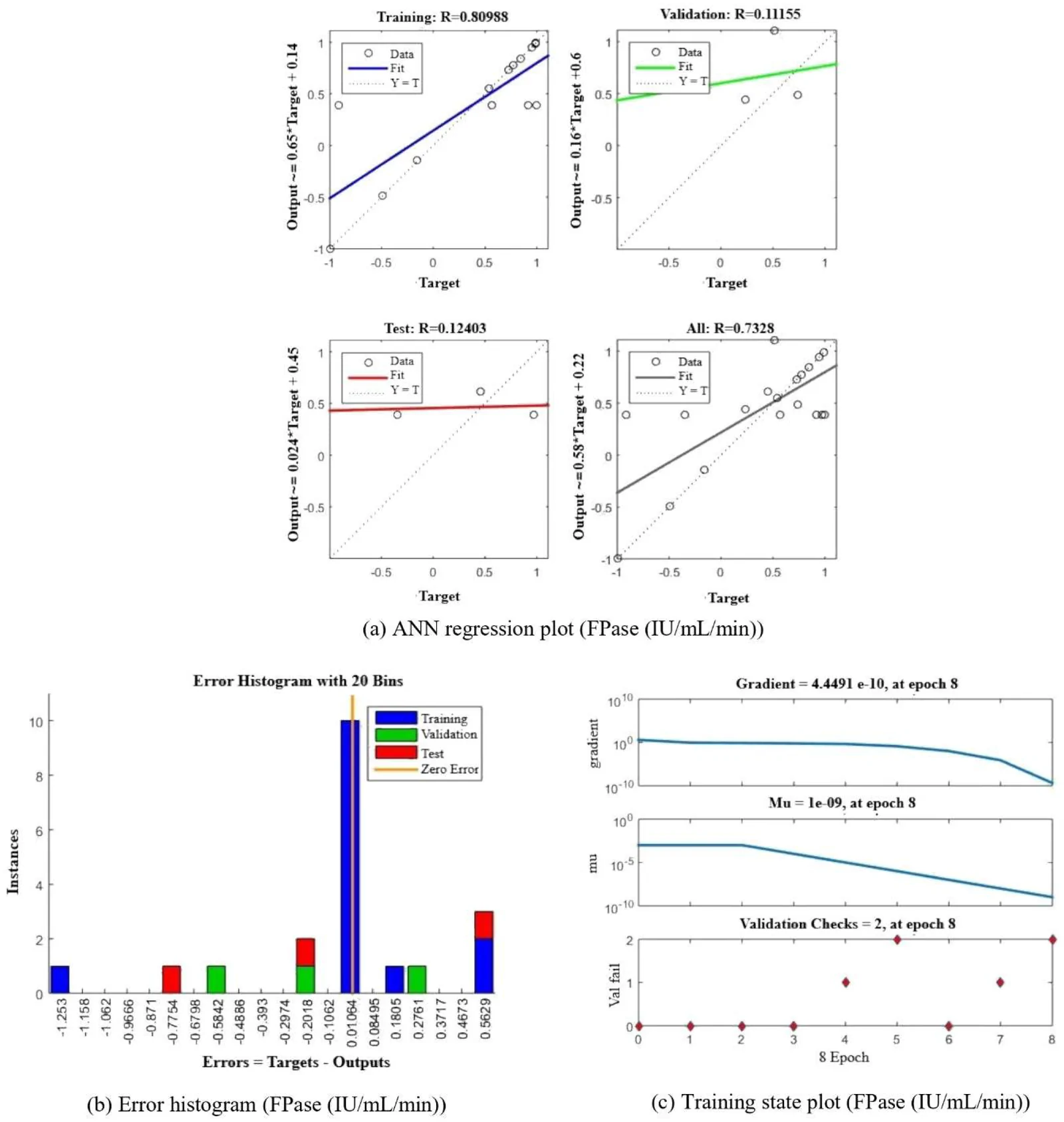
(a) Fitness of training,validation, test, and all datasets in the ANN model for FPase optimization, (b) Error histogram, and (c) Training state plot (FPase (IU/mL/min)).

The numerical records for CMCase and FPase neural networks, including Quantities, Training MSE, Validation MSE, Testing MSE, Training Time, Gradient, Performance Mu, Epoch, RMSE, and R^2^, are given in Table 6. The statistical models were evaluated using their internal goodness-of-fit and prediction statistics.

**Table 6.** Numerical results for CMCase and FPase of the ANN model.

Quantities	Training MSE /e−2	Validation MSE /e−3	Testing MSE /e−2	Training Time	Gradient /e−11	Performance /e−2	Mu /e−7	Epoch	RMSE	R^2^
CMCase (IU)	2.440165	8.524963	7.797103	0.2030	7.446639	1.360395	1000	5	1.7137	91.44%
FPase (IU)	16.97913	155.1303	30.07254	0.1560	44.49053	16.97577	1.000	6	19.1471	53.70%

### 4.3. CMCase and FPase sensitivity analysis

Sensitivity analysis of CMCase and FPase with respect to peptone levels are shown in Figures 6 and 7, respectively. According to this, CMCase sensitivity was higher for peptone, but it was lower for yeast extract and iron sulphate. In the case of FPase, there was a significantly different response. Sensitivity to yeast extract became significantly higher, indicating that yeast extract was the most significant variable determining the level of FPase. The sensitivity was negative for iron sulphate in the case of FPase. A similar interpretation style was followed for the remaining plots. In general, the sensitivity analysis revealed that CMCase production was affected mostly by the peptone levels, while the FPase production was largely determined by the level of yeast extract. The desirability chart analyses for CMCase and FPase are shown in Figure 8. The difference between observed and predicted values for CMCase, RSM (left) and ANN (right), are shown in Figure 9. The difference between observed and predicted values for FPase, RSM (left) and ANN (right), are shown in Figure 10.

**Figure 6.**
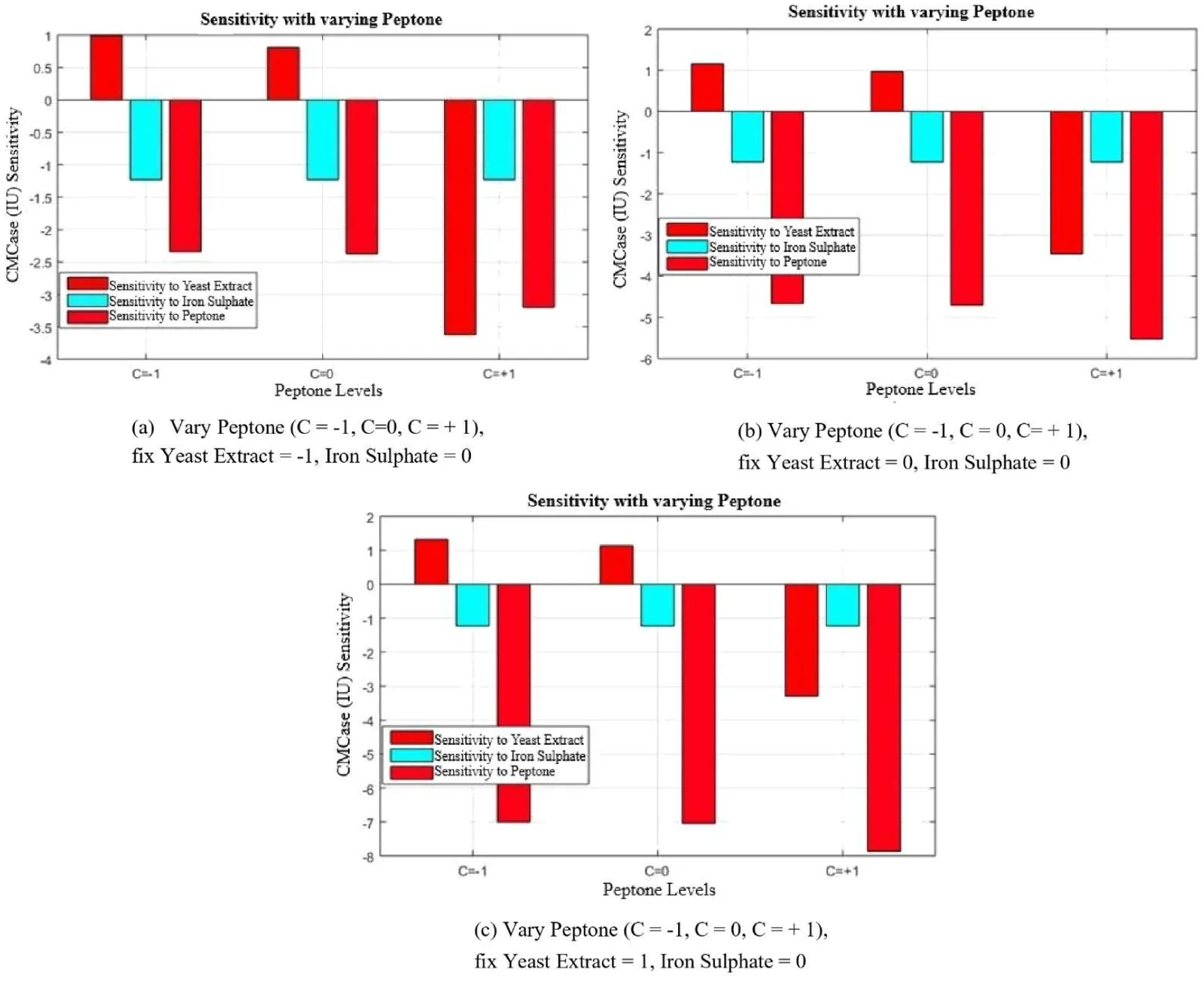
Sensitivity analysis of CMCase with respect to the peptone level.

**Figure 7.**
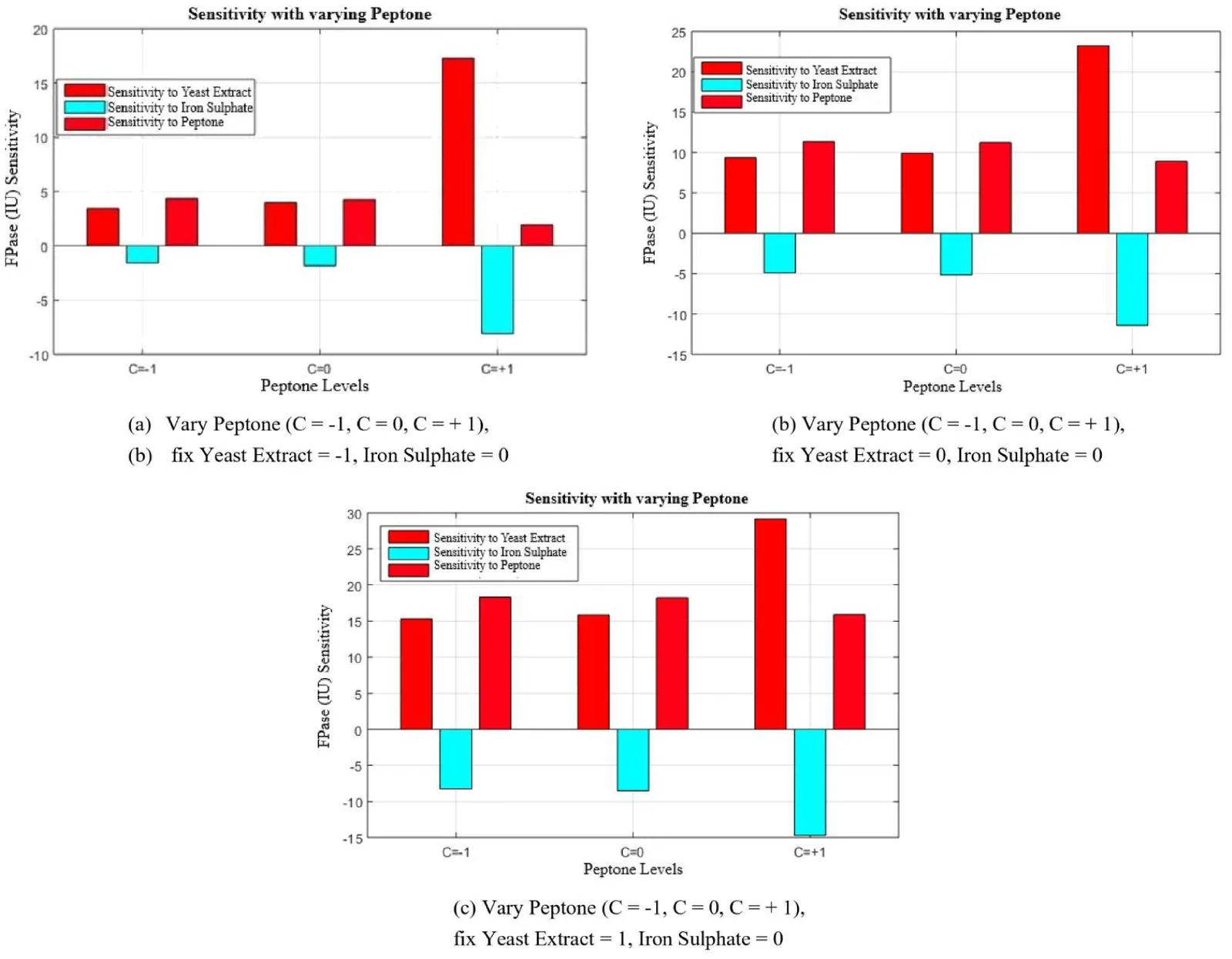
Sensitivity analysis of FPase with respect to Peptone level.

**Figure 8.**
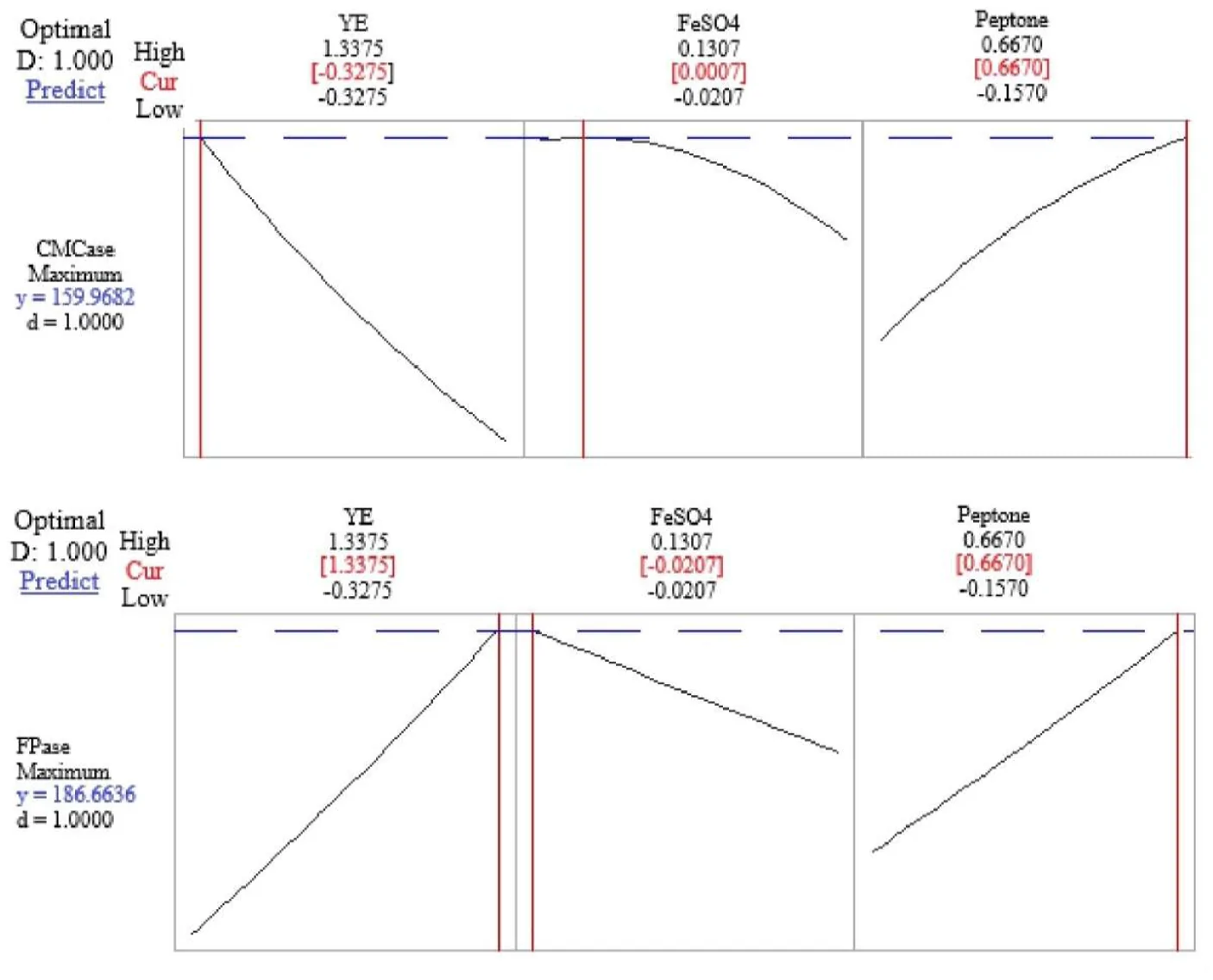
Desirability chart analysis of CMCase and FPase.

**Figure 9.**
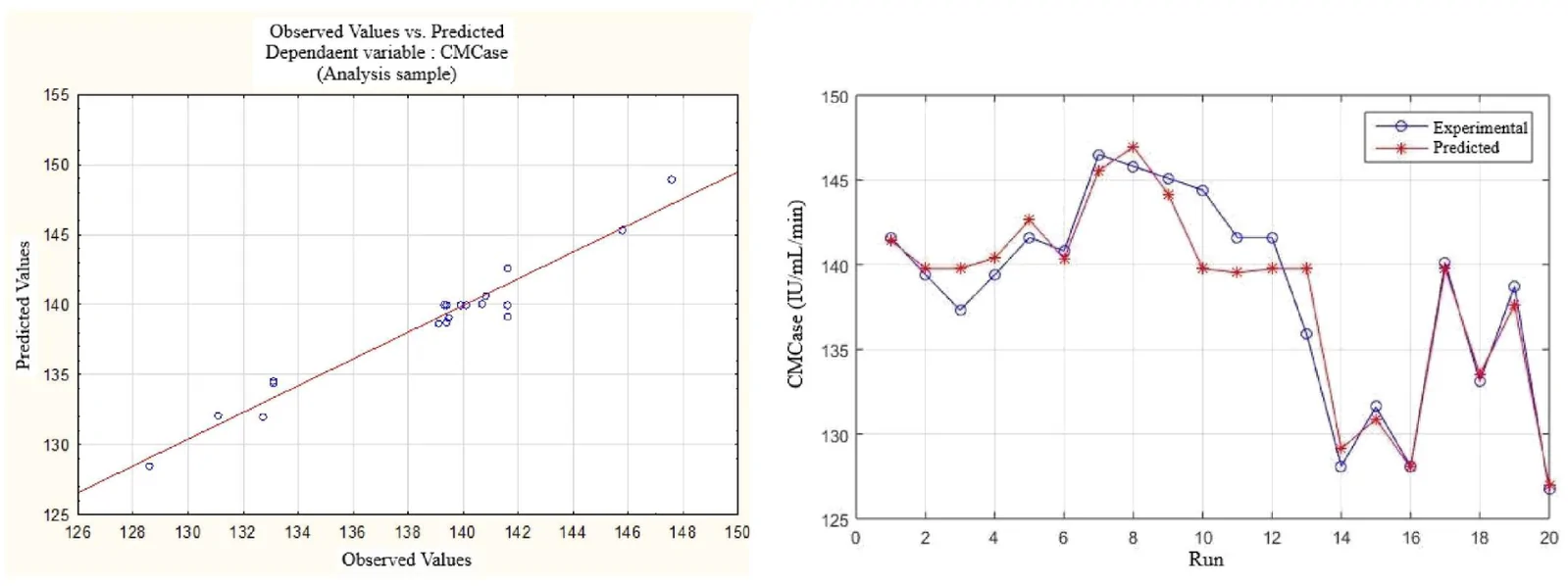
Difference between observed and predicted values of CMCase; RSM (left) and ANN (right).

**Figure 10.**
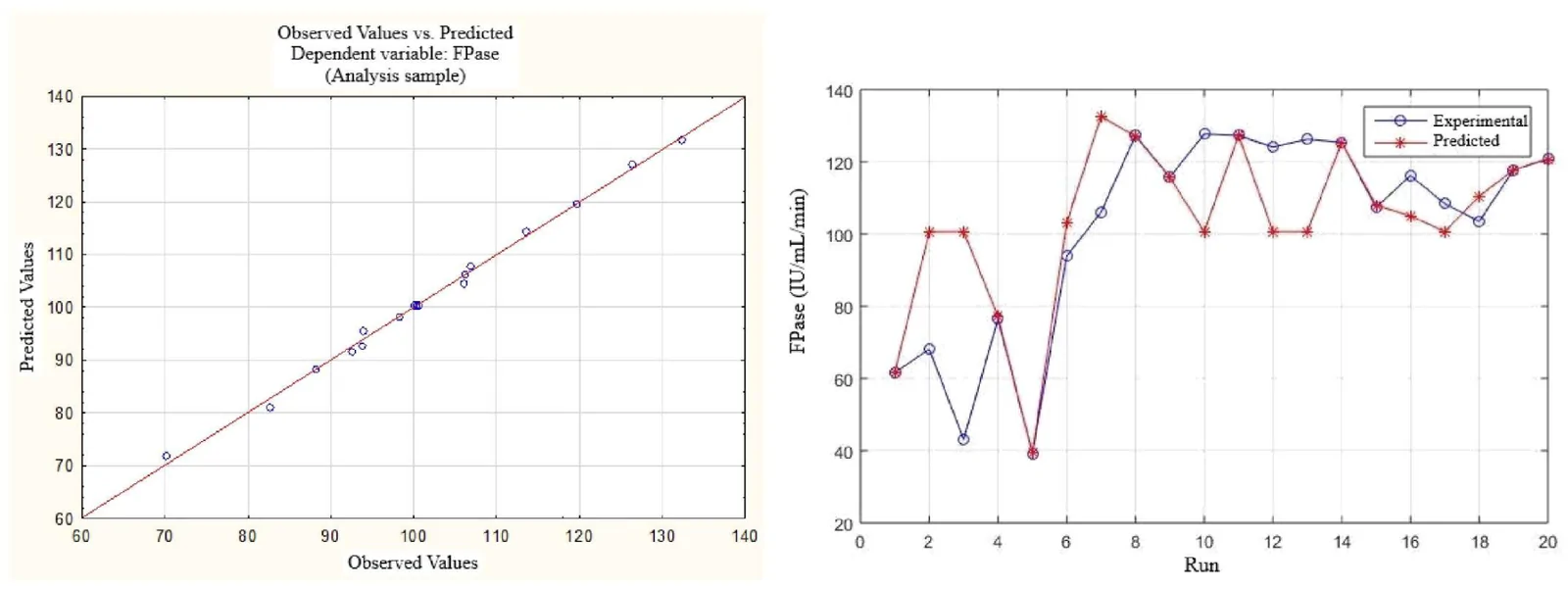
Difference between observed and predicted values plot of FPase; RSM (left) and ANN (right).

The regression equation used for CMCase is:

CMCase (IU) = 138.84 − 2.63 YE − 27.0 FeSO_4_ + 29.87 Peptone + 3.85 YE × YE + 815 FeSO_4_ × FeSO_4_ − 19.04 Peptone × Peptone + 98.2 YE × FeSO_4_ − 38.45 YE × Peptone + 91.8 FeSO_4_ × Peptone

The regression equation used for FPase is:

FPase (IU) = 88.13 + 0.42 YE + 402.6 FeSO_4_ − 41.21 Peptone + 5.27 YE × YE + 1 FeSO_4_ × FeSO_4_ + 11.22 Peptone × Peptone − 298.0 YE × FeSO_4_ + 115.34 YE × Peptone 595.2 FeSO_4_ × Peptone

In this analysis, X = yeast extract, Y = FeSO_4_, and Z = Peptone, and the results in Table 7 were obtained from the calculations below. Table 7 indicates the coded coefficients of CMCase and FPase. The sensitivity analysis was done through differentiation of the variables. The coefficients having p-values of less than 0.05 were statistically significant and used for sensitivity analysis. Therefore, the sensitivity analysis required coded coefficients with significant p-values, which were calculated using the Minitab software.

**Table 7.** Coded coefficients of CMCase and FPase with significant p-values.

Term	CMCase		FPase
Term	Coefficients	P-Value	Coefficients	P-Value
Constant	139.984	0.000	100.287	0.000
Yeast extract	−3.116	0.002	9.293	0.000
FeSO_4_	−3.927	0.000	4.520	0.000
Peptone	2.840	0.004	−2.445	0.000
YE × YE	1.887	0.028	1.292	0.003
FeSO_4_ × FeSO_4_	−3.300	0.001	0.002	0.996
Peptone × Peptone	−2.285	0.011	0.673	0.071
YE × FeSO_4_	4.375	0.001	−6.638	0.000
YE × Peptone	−9.325	0.000	13.988	0.000
FeSO_4_ × Peptone	2.025	0.068	6.562	0.000

The mathematical form of the regression model can be defined as:

CMCase = β_0_ + β_1_ × X + β_2_ × Y + β_3_ × Z + β_4_ × X × X + β_5_ × Y × Y + β_6_ × Z × Z + β_7_ × X × Y + β_8_ × X × Z + β_9_ × Y × Z

Based on the p-value, we can write the expression of regression as:

CMCase = 139.984 − 1.558×X − 1.963 × Y + 1.420 × Z + 0.943 × X × X − 1.650 × Y × Y − 1.143 × Z × Z + 2.187 × X × Y − 4.663 × X × Z

The sensitivity analysis can be calculated by taking derivatives of the above expression as:

$$\frac{\partial (CMCase)}{\partial X}=-1.558X+1.886X+2.187Y-4.663Z$$

$$\frac{\partial (CMCase)}{\partial Y}=-1.963Y-3.3Y+2.187Y$$

$$\frac{\partial (CMCase)}{\partial Z}=1.420Z-2.286Z-4.663X$$

The mathematical form of the regression model can be defined as:

FPase = β_0_ + β_1_ × X + β_2_ × Y + β_3_ × Z + β_4_ × X × X + β_5_ × Y × Y + β_6_ × Z × Z + β_7_ × X × Y +β_8_ × X × Z + β_9_ × Y × Z

Based on the p-Value, we can write the expression of regression as:

FPase = 100.287 + 9.293 × X + 4.520 × Y − 2.445 × Z + 1.292 × X × X − 6.638 × X × Y + 13.988 × X × Z − 6.562 × Y × Z

The sensitivity analysis can be calculated by taking derivatives of the above expression as:

$$\frac{\partial (FPase)}{\partial X}=9.293X+2.584X-6.638Y+13.988Z$$

$$\frac{\partial (FPase)}{\partial Y}=4.520Y-6.638X-6.562Z$$

$$\frac{\partial (FPase)}{\partial Z}=-2.445Z+13.988X-6.562Y$$

### 4.4. Enzyme characterization analysis

#### 4.4.1. Effects of temperature

In this study, the impact of temperature, ranging from 30 °C to 90 °C, on the activity and stability of CMCase and FPase enzymes was evaluated. The results illustrated how varying temperatures influenced the enzymatic performance of CMCase and FPase, as illustrated in Figure 11. During enzyme stability analysis, samples were placed at selected temperatures for 120 minutes and were taken out every 20 minutes to check their CMCase and FPase activities.

**Figure 11.**
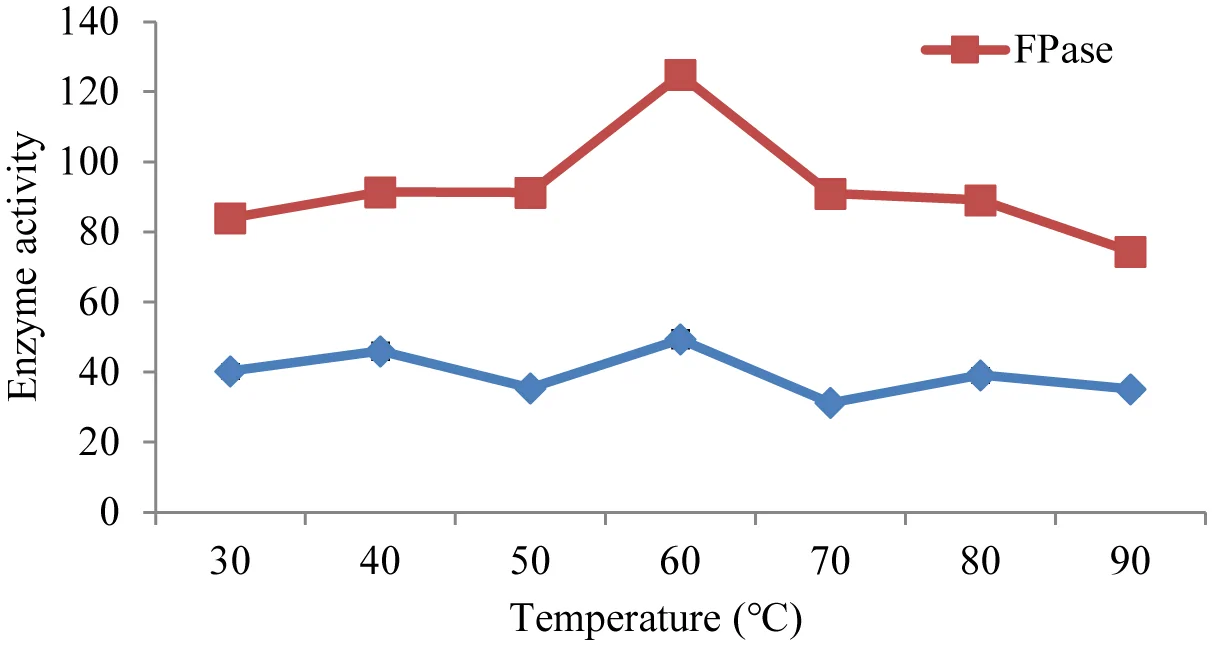
Effect of temperature on cellulolytic activity of the enzyme.

Results of the effect of temperature on CMCase and FPase stability are shown in Figure 12. The maximum CMCase and FPase activities were observed at 60 °C. The higher optimum temperature observed in this study compared with several *Bacillus cellulases* reported at 40–50 °C may reflect strain-specific differences in enzyme structure and thermostability [39]. Similarly, FPase and CMCse showed significant stability at 30 °C to 100 °C temperature analyses [40]. Such broad thermal stability is particularly advantageous for industrial applications and is consistent with the reported thermostability of cellulases from some *Bacillus* strains [41]. Moreover, for *B. subtilis*, the optimum cellulose temperature for CMCase activity was around 40 °C [42]; *B. subtilis* has shown maximum activity of cellulase produced by cellulase-producing bacteria in molasses at 40 °C [43]. Furthermore, *Bacillus amyloliquefaciens* exhibited an optimum temperature for maximum stability and activity of cellulase at 50 °C. Above this temperature, the activity declined, and significantly low activity was observed at 90 °C.

**Figure 12.**
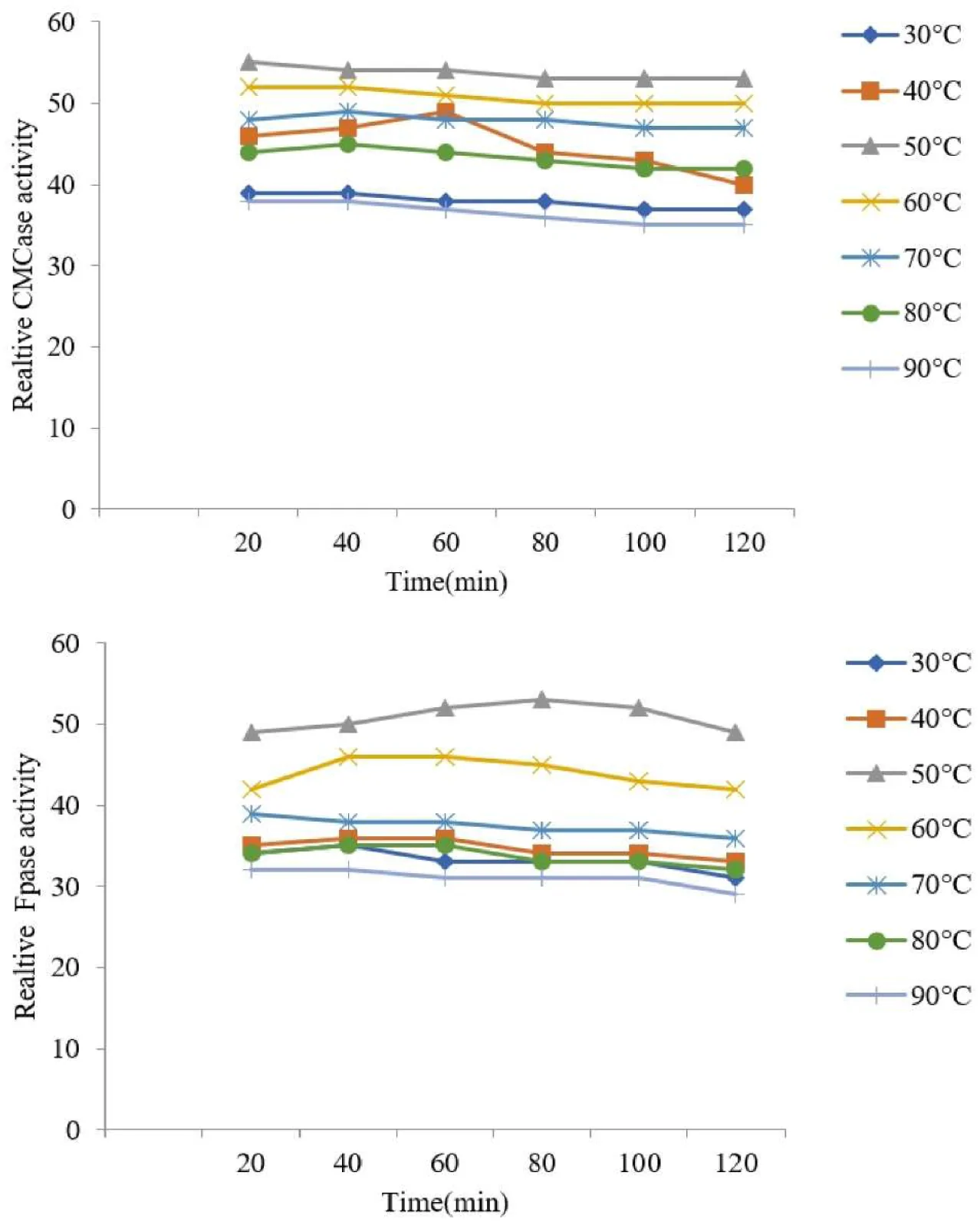
Stability of the cellulolytic enzyme at varying temperatures.

#### 4.4.2. Effects of pH

In this investigation, the effect of pH ranges from 4 to 9 for CMCase and Fpase activities and the stability were analyzed (Figure 13). During enzyme stability analysis, the samples were placed at selected pH values for 120 minutes and were removed to check their CMCase and FPase activities. In this study, maximum CMCase and FPase activities were achieved at pH 6.0; enzyme stability analysis demonstrated that CMCase and FPase remained stable at pH 6.0 [44]. The stability of both enzymes at pH 6.0 suggests that the strain produces cellulases adapted to mildly acidic conditions, which may be beneficial for processes operating near this pH [45]. Similarly, cellulase from strains of *Streptomyces macrosporeus* have shown maximum activity after 120 h of incubation at pH 6.0 and 2% CMC [46]. Moreover, *B. subtilis* CD001 showed maximum enzyme activity at pH 7.0. Furthermore, the pH range for maximum cellulase activity from *Bacillus* sp. and *Pseudomonas* sp. is 7.5 to 8.0 [47]. Additionally, *B. subtilis* YJ1 showed that the optimal pH for cellulase synthesis was between 6 and 8, as S1-1, S7-2, and S7-6 showed peak production at pH 6.0, S1-4 showed maximum cellulase production at pH 7.0, and S3-3 and S4-2 showed optimum cellulase production at pH 8.0.

**Figure 13.**
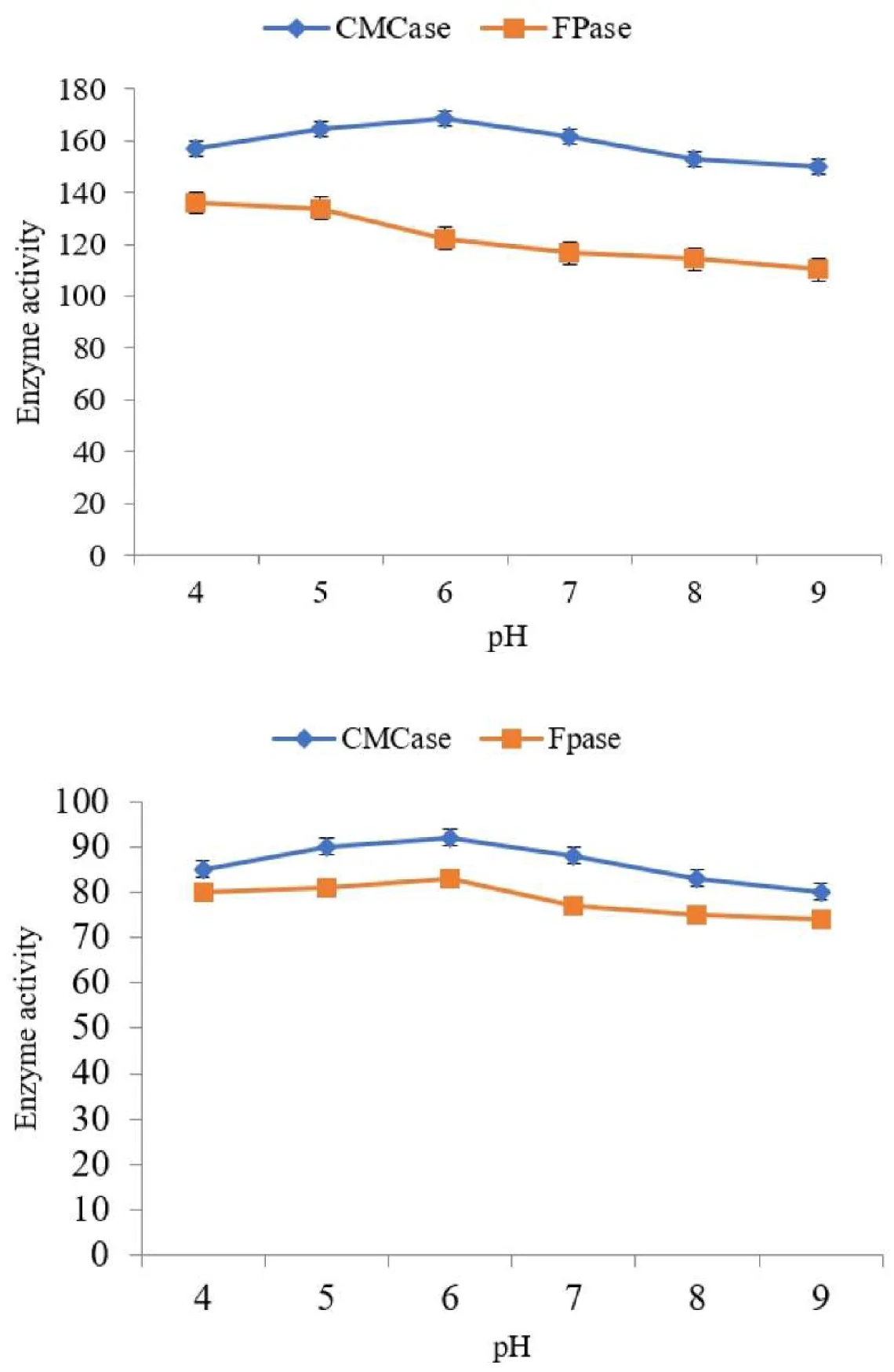
Effect of pH on CMCase and FPase activities (top) and stability (below).

#### 4.4.3. Effects of metal ions

In this research, 5 mM solutions of various metal ions, including CaCl_2_, KCl, MnSO_4_, MgSO_4_, FeSO_4_, CuSO_4_, EDTA, and NaCl, were used to check their effects on enzyme activity and stability. A single concentration was chosen to create a screening condition. This made it possible to compare how the tested additives affected the enzyme under the experimental settings. For stability testing, the crude enzyme was first mixed with each additive separately and incubated at room temperature for 120 minutes. After this time, the remaining CMCase and FPase activities were measured using the assay methods.

The results were shown as percentages compared to the control that had no additive. Maximum CMCase relative activity (74.7%) was achieved at 5 mM concentrations of CaCl_2_, while maximum FPase relative activity (76.9%) was obtained at 5 mM concentrations of FeSO_4_. Moreover, the effect of other metal ion solutions for CMCase and FPase activities and stabilities are shown in Figure 14 [48].

The tested metal ions affected CMCase and FPase activities to different extents, with enzyme activities generally lower than the control, indicating that these additives did not enhance cellulase activity under the tested conditions. *Bacilius vallismortis* has shown maximum cellulase activity with CaCl_2_ [49]. In the literature, *Bacillus subtilis* SV1showed that Zn^2+^, Co^2+^, and Fe^2+^ were favorable for cellulase activity [50]; however, Mg^2+^ and Ca^2+^ had positive effects on the activities of endoglucanase and exoglucanase.

**Figure 14.**
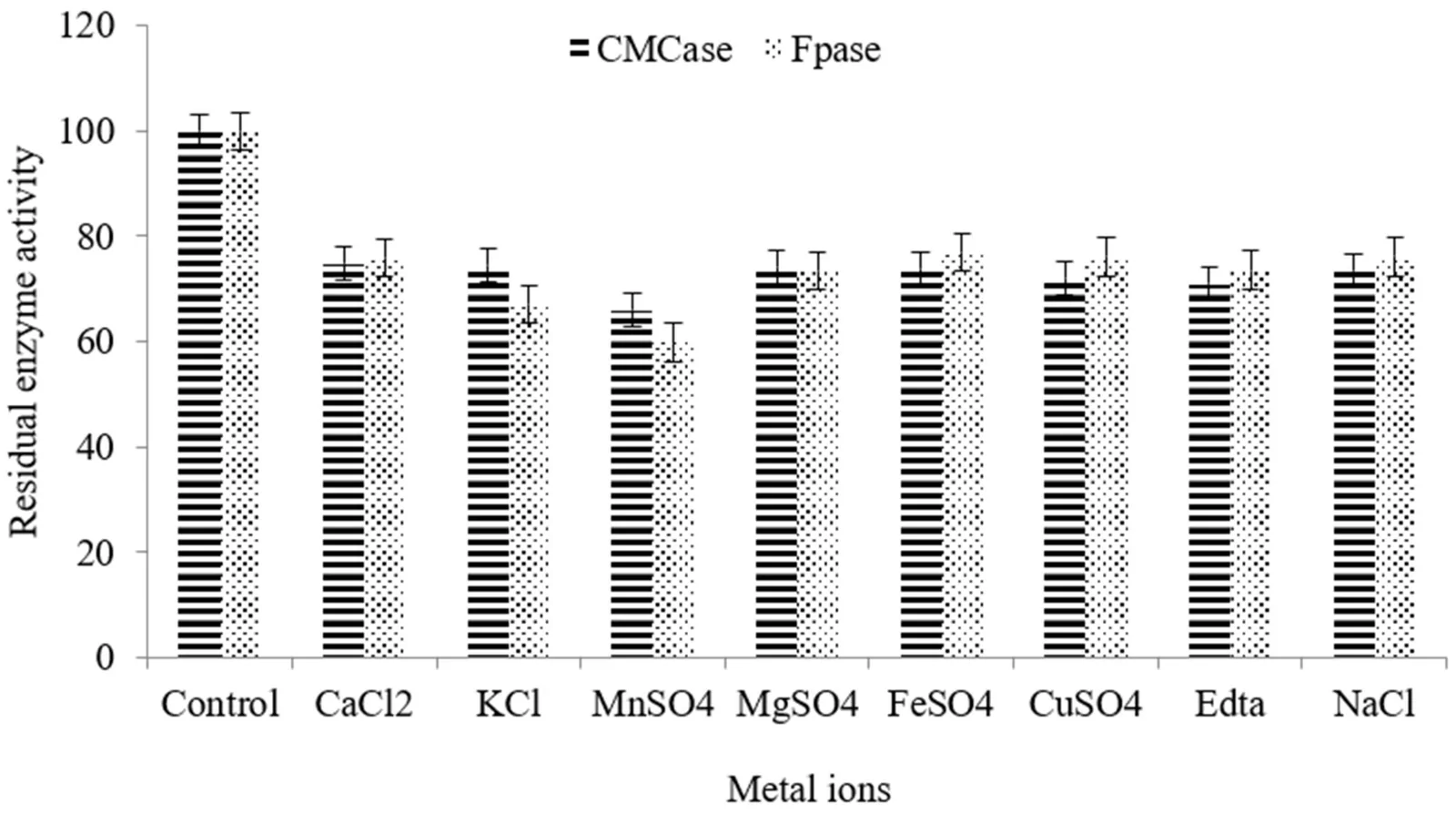
Effect of metal ions on cellulolytic enzyme activity and stability.

#### 4.4.4. Effects of solvents

Moreover, six solvents, including isopropanol, methanol, ethanol, butanol, acetone, and chloroform, were studied to identify their effects on CMCase and FPase stabilities and activities. We used a screening concentration of 5 mM. For the stability analysis, the enzyme was pre-incubated separately with each solvent for 120 minutes before measuring the enzymatic activities. The enzyme solution without solvent treatment was used as a control. The organic solvents tested in this study generally reduced CMCase and FPase activities compared with the control, as shown in Figure 15. Maximum CMCase relative activity (74.1%) and maximum FPase relative activity (78%) was achieved at 5 mM concentration of ethanol solvent. *B. subtilis* SU40 and *Bacillus cellulose* have shown to be affected by solvent for CMCase and FPase, respectively [27].

**Figure 15.**
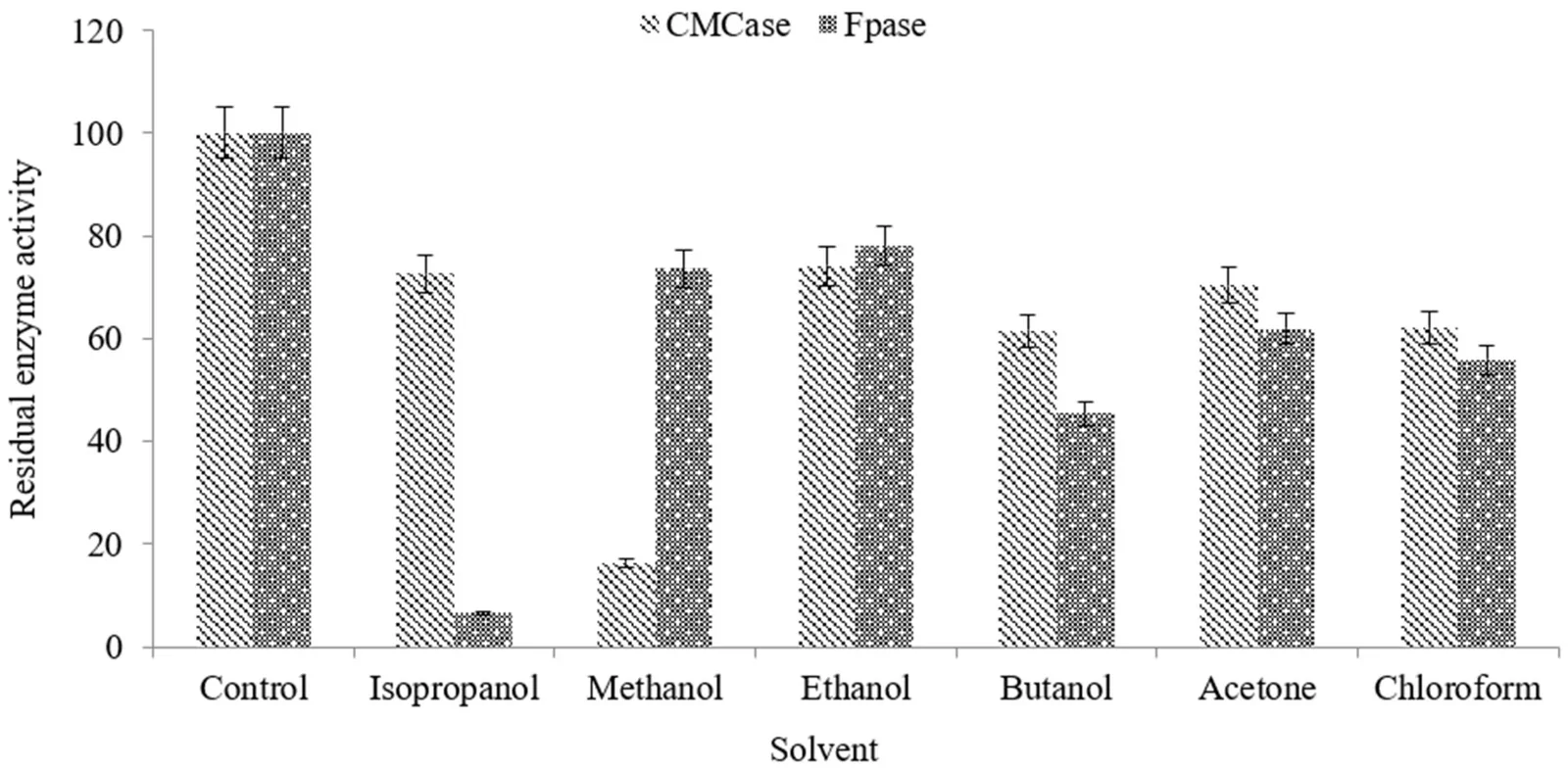
Effects of solvents on cellulolytic enzyme stability.

#### 4.4.5. Surfactant and detergent analysis

Moreover, four surfactants (Tween-80, Tween-20, Triton X-100, and SDS) were studied to check their effects on CMCase and FPase stabilities. Maximum CMCase and FPase relative activities were achieved at 5 mM concentration of SDS and Tween-20, as shown in Figure 16. The enhanced activity was observed with SDS and Tween-20, contrasting with the inhibitory effect of SDS reported for some *Bacillus* cellulases, suggesting strain-specific differences in surfactant tolerance and enzyme-surfactant interactions [51],[52]. *B. subtilis* M11 has shown minimum cellulase activity with SDS and sometimes with Tween-80 [53].

Moreover, detergents used in this research included Brite, Surf Excel, Express, and Tap, as shown in the Figure 17. The cellulolytic enzymes worked well with detergents. The CMCase activity was highest with Sooraj at 115% and lowest with Moli at around 70%. For FPase activity, the highest level was seen with Express at 105%, while Saroo showed the lowest at 70%.

These results match what has been reported, where microbial cellulases have shown stability in detergent environments. For example, *Bacillus sp*. PCH94 β-1,4-glucanaseshowed that it kept more than 85% of its activity when exposed to commercial detergents [54]. Similarly, *Bacillus pumilus* VLC7 cellulase maintained 92 to 98% activity in detergents [55]. Overall, most detergents maintained considerable enzyme activity, indicating good stability and compatibility with detergents. However, bacterial cellulases remained stable in the surfactants applied, including SDS, Tween-20, 40, and 80 [56].

**Figure 16.**
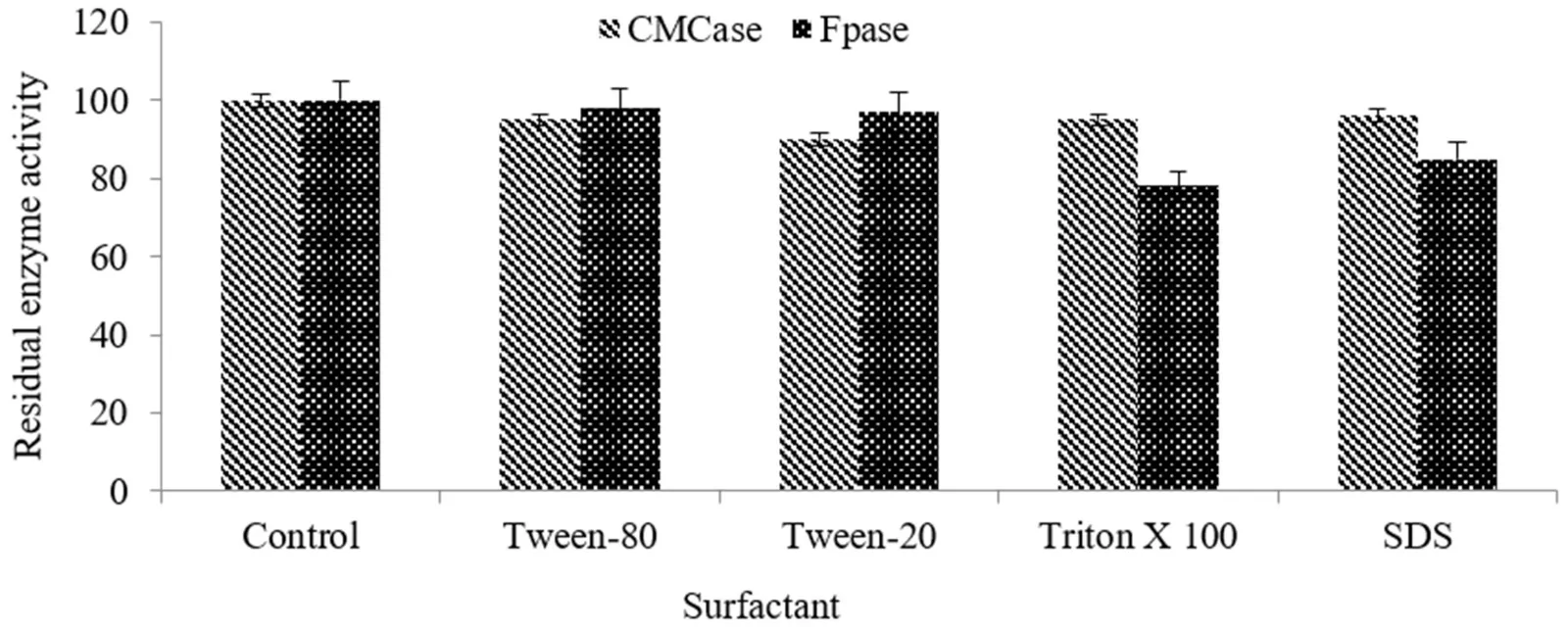
Effects of surfactants on cellulolytic enzyme activity and stability.

**Figure 17.**
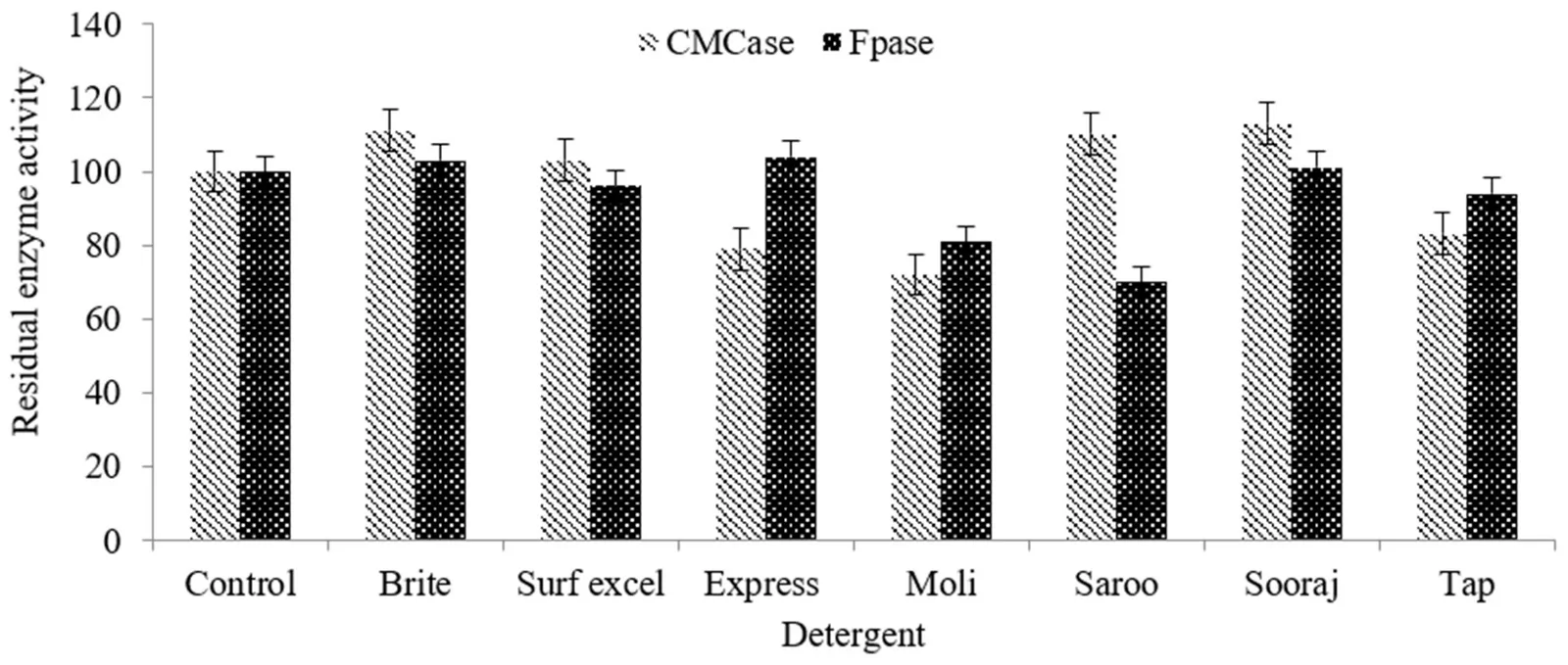
Effects of detergents on cellulolytic enzyme activity and stability.

#### 4.4.6. Thermodynamics evaluation

Arrhenius analysis was conducted o identify how temperature affects CMCase and FPase activities within the range that was studied as shown in Figure 18. The apparent activation energy (Ea) was calculated using the slope from the linearized Arrhenius plot. ln(v) = ln f_0_ (A) − Ea/RT, where v stands for the measured enzyme activity, A is the pre-exponential factor, R is the universal gas constant, and T is the absolute temperature. Accordingly, Ea was calculated from the slope of the Arrhenius plot. The recalculated apparent activation energies were 16.66 kJ·mol^−1^ for CMCase and 7.35 kJ·mol^−1^ for FPase. Additionally, the enthalpy change (∆H) for CMCase and FPase were 14.08 kJ·mol^−1^ and l4.78 kJ·mol^−1^, respectively. Moreover, entropy (∆S) for CMCase and FPase were −40 J·mol^−1^·K^−1^ and −20 J·mol^−1^·K^−1^, respectively. Consequently, the calculated thermodynamic parameters were interpreted cautiously and were not used to draw definitive mechanistic conclusions [57].

**Figure 18.**
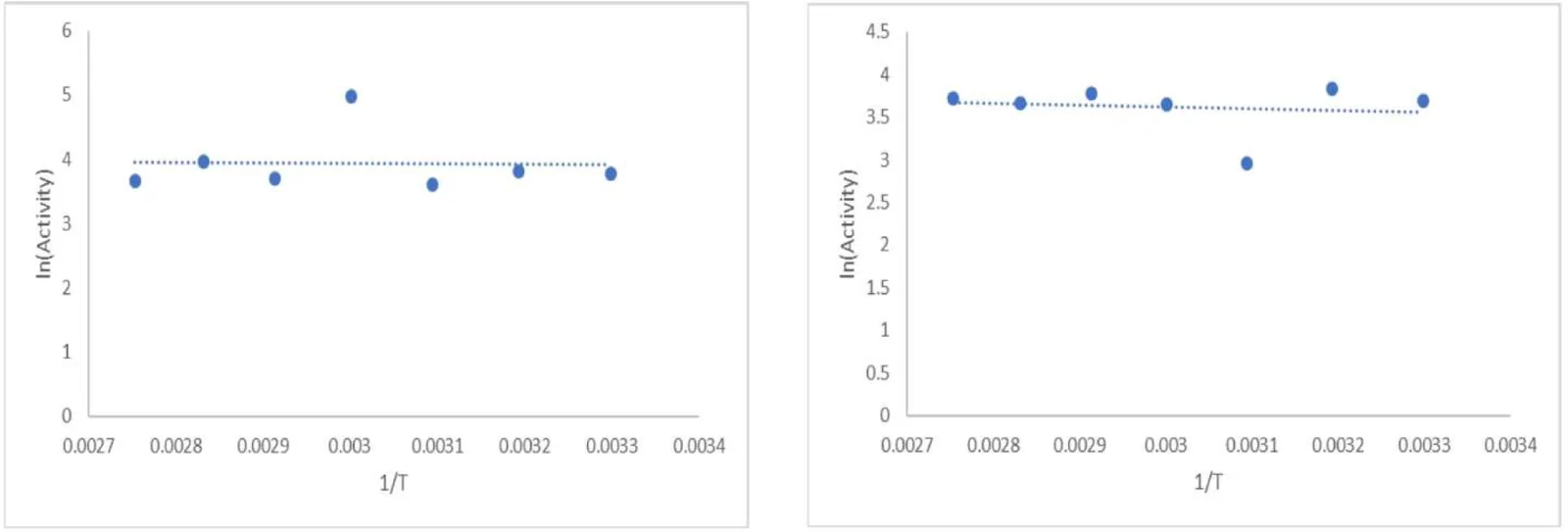
Arrhenius plot analysis for CMCase (left) and FPase (right).

## 5. Conclusions

Enzymes are biological catalysts that have increasingly replaced chemical catalysts due to rising environmental concerns and the demand for sustainable technologies. Cellulose, the most abundant natural polymer, is a major structural component of plant cell walls. Cellulase enzymes play a crucial role in hydrolyzing this complex polysaccharide into simple sugar units. This study was designed to isolate, purify, and characterize novel *Bacillus* strains with enhanced cellulase-producing potential.

Exoglucanase production by the selected strains was optimized using RSM (RSM) and ANN (ANN). Thus, the R^2^ value calculated by RSM analysis for CMCase was 95.43% and for FPase was 99.58%. The R^2^ values calculated by ANN analysis for CMCase was 91.44% (Overall R = 0.95622), and for FPase was 53.70% (Overall R = 0.7328). Thus, the RSM and ANN models provided different predictive performances for CMCase and FPase. Initially, the PBD was employed to screen the most influential factors affecting enzyme production. Subsequently, the CCD was applied for precise optimization of these critical parameters.

Furthermore, detailed enzyme characterization was conducted, including the assessment of thermal stability, pH tolerance, and the influence of metal ions on enzymatic activity. The tested metal ions affected CMCase and FPase activities to varying degrees but did not enhance enzyme activity compared with the control. Similarly, the tested organic solvents generally decreased enzyme activities, although the extent of inhibition varied depending on the enzyme and solvent. The production of endoglucanase and exoglucanase from *Bacillus velezensis* 2H was successfully optimized using RSM under submerged fermentation conditions. Moreover, the carrot, an inexpensive yet nutritionally rich substrate, significantly enhanced cellulase production. Furthermore, characterization of the produced cellulases depicted their activities and stabilities under varying environmental conditions, highlighting their potential for industrial applications.

## Use of AI tools declaration

AI tools were not used in the preparation of the manuscript.
